# Profiling RNA editing in human tissues: towards the inosinome Atlas

**DOI:** 10.1038/srep14941

**Published:** 2015-10-09

**Authors:** Ernesto Picardi, Caterina Manzari, Francesca Mastropasqua, Italia Aiello, Anna Maria D’Erchia, Graziano Pesole

**Affiliations:** 1 Department of Biosciences, Biotechnology and Biopharmaceutics, University of Bari, Via Orabona 4, 70126 Bari, Italy; 2 Institute of Biomembranes and Bioenergetics, National Research Council, Via Amendola 165/A, 70126 Bari, Italy; 3 National Institute of Biostructures and Biosystems (INBB), Viale Medaglie D’Oro 305, 00136 Rome, Italy; 4 Center of Excellence in Comparative Genomics, University of Bari, Piazza Umberto I, 70121 Bari, Italy

## Abstract

Adenine to Inosine RNA editing is a widespread co- and post-transcriptional mechanism mediated by ADAR enzymes acting on double stranded RNA. It has a plethora of biological effects, appears to be particularly pervasive in humans with respect to other mammals, and is implicated in a number of diverse human pathologies. Here we present the first human inosinome atlas comprising 3,041,422 A-to-I events identified in six tissues from three healthy individuals. Matched directional total-RNA-Seq and whole genome sequence datasets were generated and analysed within a dedicated computational framework, also capable of detecting hyper-edited reads. Inosinome profiles are tissue specific and edited gene sets consistently show enrichment of genes involved in neurological disorders and cancer. Overall frequency of editing also varies, but is strongly correlated with ADAR expression levels. The inosinome database is available at: http://srv00.ibbe.cnr.it/editing/.

Large-scale projects such as ENCODE have shed light on the dynamic nature of eukaryotic transcriptomes, where diverse molecular processes interact to fine-tune gene expression^1^. Among them, the co- and post-transcriptional RNA editing plays a pivotal role as it may expand the molecular diversity of the transcriptomes and, consequently, the proteomes of a wide range of organisms^2^. RNA editing affects thousands of primary transcripts through the insertion, deletion or substitution of certain nucleotides at specific RNA locations^3^.

In humans, the most prevalent type of RNA editing converts adenosine (A) residues into inosine (I) in double stranded RNAs through the deamination reaction carried out by members of the adenosine deaminase (ADAR) family of enzymes, comprising ADAR1 (known also as ADAR), ADAR2 (known also as ADARB1) and ADAR3 (known also as ADARB2)^4^. While ADAR1 and ADAR2 are expressed in almost all human tissues to a greater or lesser extent, ADAR3 is brain specific and its catalytic activity remains unknown (Supplementary Figs 1, 2 and 3).

Inosine is commonly interpreted as guanosine by the cellular machinery (and sequencing enzymes) leading to functional consequences including the alteration of codon identity, the creation or elimination of splice sites and the modification of base-pairing interactions within higher-order RNA structures^4^. RNA editing has a critical role in cellular homeostasis and a variety of human disorders such as schizophrenia, major depression, amyotrophic lateral sclerosis and cancer are linked to its deregulation^5^,^6^. In mice, the selective knockout of ADAR1 or ADAR2 causes extremely severe phenotypes^7^,^8^.

High-throughput sequencing platforms and effective computational technologies have facilitated transcriptome-wide studies of RNA editing^9^,^10^. According to the RADAR database (a collection of rigorously annotated A-to-I events), about 2,5 million sites in the human transcriptome undergo RNA editing and more than 95% of these positions resides in Alu repetitive elements. Such repeats are widespread in human genes (they account for around 10% of the genome), are frequently transcribed, and often form double stranded RNA structures^11^. The vast majority of known RNA editing sites were detected by computational methods that meticulously compare genomic and transcriptomic sequences from the same individual, incorporating *ad hoc* filters to minimize false candidate calls due to sequencing or read mapping errors^9^,^10^,^11^,^12^. Several of these methods have been applied to cell lines, including lymphoblastoid cell lines (LCLs)^9^,^10^,^11^,^12^,^13^, which may not be entirely representative of physiological situations. Indeed, LCLs, obtained by the transformation of peripheral B-lymphocytes by Epstein-Barr virus, show atypically high and low expression levels for ADAR and ADARB1 enzymes, respectively (FPKM of 126.42 for ADAR and 0.16 for ADARB1 [n = 54]) when compared to different primary tissue types^14^ (Supplementary Figs 1 and 2). Here we present a comprehensive human RNA editing atlas generated through comparisons of matched genomic and transcriptomic data, across six tissues (brain, lung, kidney, liver, heart and muscle) from three post-mortem healthy individuals (sex, age and race matched with post-mortem delay less than 5 hours) (Table 1).

Distinct from other large-scale studies^15^,^16^,^17^, we have employed sampling and sequencing strategies dedicated to the study of RNA editing, producing on average 167 millions of paired and strand oriented RNA reads per tissue and resequencing the whole exome and genome of each individual (Supplementary Table 1). The use of an improved computational strategy incorporating a recent approach for the detection of hyper-edited reads^18^, allowed the identification of a total of 3,041,422 A-to-I changes including over 2 million novel events.

## Results

### RNA editing detection

Strand-specific RNA-Seq reads were subjected to stringent quality trimming and aligned to the reference human genome using the spliced aligner GSNAP^19^, which has been shown to identify more usable reads than BWA or bowtie^20^.

**Figure 1:**
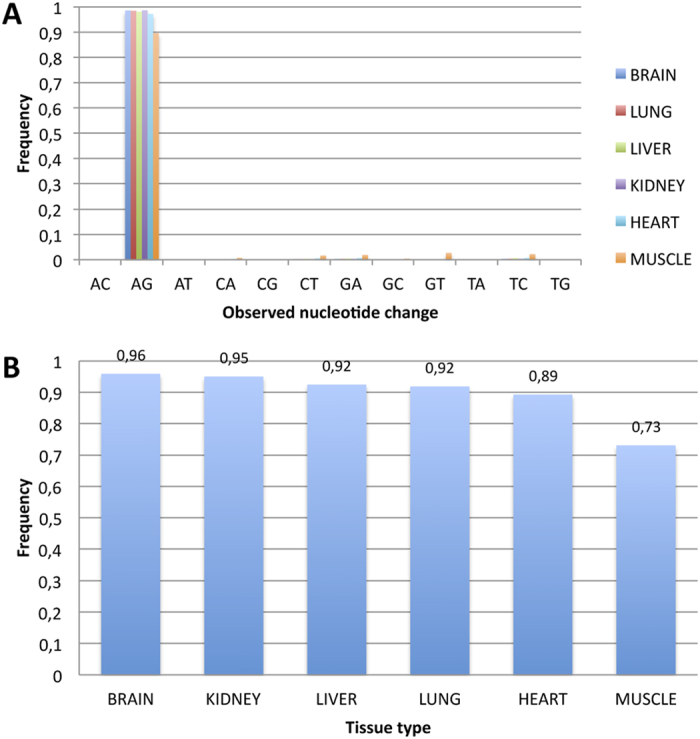
Frequencies of observed nucleotide changes. (**A**) Most of the detected RNA editing events were A-to-G. Potential non canonical events were rare and showed frequency values less than 0.05. (**B**) The fraction of A-to-G changes in non-synonymous sites across tissues.

The impact of misalignments was mitigated by applying Blat, a more accurate aligner than fast-mappers like GSNAP, on reads carrying mismatches^9^. Contrary to previous methods, only read pairs providing unique genome mapping locations were considered. RNA editing candidates were called using the REDItools package^20^, and considering only sites that were homozygous in the genomic DNA, obtained by whole genome resequencing of the same individual. In case of positions falling in non-repetitive regions for which the RNA editing detection is challenging, we applied stringent filters excluding DNA-RNA changes in the first and last 6 bases of reads^9^, in homopolymeric regions longer than 5 residues, located near splice sites, variants supported by less than 3 reads and surrounding genomic regions in which the multiple alignment of reads was not optimal by the presence of indels (see Methods). The genome-wide screening of RNA editing in all six tissues yielded a total of 2,013,010 A-to-I events with a supposedly high specificity as 97% of all DNA-RNA changes were of A-to-G type^18^ (Fig. 1A). Indeed, it is well known^18^ that the higher the enrichment of A-to-G changes, the higher the A-to-I editing detection specificity, particularly in coding regions. Notably, we registered a much higher A-to-G occurrence in nonsynonymous sites (on average 89.5%, ranging from 73% in muscle to 96% in brain) (Fig. 1B) than previous studies (<40%)^9^,^12^, thus confirming the accuracy of our methodology.

The concomitant use of strand oriented RNA-Seq reads and whole genome sequences, in combination with a refined computational methodology, allowed us to detect a very low fraction of apparent non-canonical RNA editing sites (Fig. 1A) which likely represent false positive calls^21^.

We also extended our screening to C-to-U editing sites, the other type of canonical RNA editing in human through the action of APOBEC1 enzyme^22^. C-to-U editing is an extremely rare event in the human transcriptome and only a few sites have been discovered up to now in physiological conditions^22^. Indeed, RNA-Seq data, showed that APOBEC1 was not expressed in any of the tissues investigated and only a handful of potential C-to-U changes (on average 0.5% of observed changes) were detected and likely correspond to false positives.

**Figure 2:**
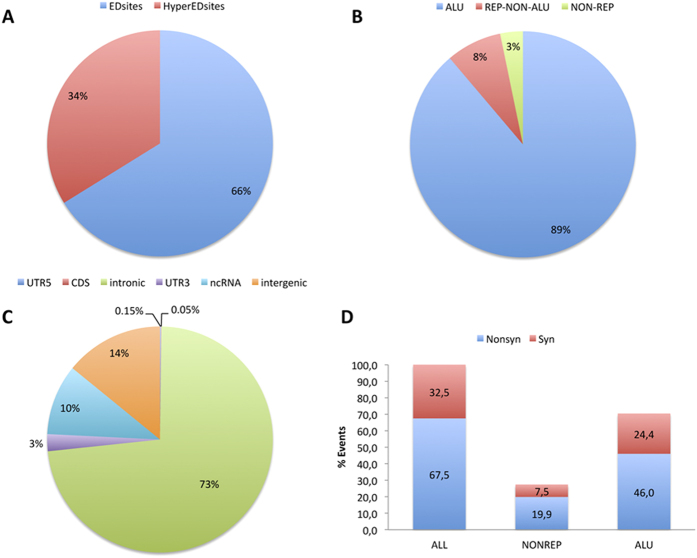
Classification of RNA editing sites. (**A**) Fraction of A-to-I sites discovered by our computational approach including events in hyper-edited reads that are currently disregarded by main RNA-Seq aligners. (**B**) Partitioning of detected RNA editing sites in Alu elements (ALU), other repetitive regions (REP-NON-ALU) and nonrepetitive regions (NON-REP). According to previous large-scale investigations, the vast majority of RNA editing sites resides in repetitive regions (97%). (**C**) Genomic localization of detected editing sites. (**D**) Fraction of synonymous and nonsynonymous A-to-I events occurring in ALU and nonrepetitive regions of open reading frames.

We also applied a recent approach to rescue heavily edited RNA-Seq reads that are generally missed by current methods^18^. After discarding positions that are heterozygous in the genomic DNA, we identified 1,028,412 hyper edited sites (Fig. 2).

Our catalogue of A-to-I editing sites was compared to the DARNED database^23^ collecting 314,250 A-to-I sites identified in older published studies and RADAR^11^ (version 2) which contains 2,576,459 events from recently published works. Overall, we found 54% overlap with DARNED and only 37% overlap with RADAR. However, we also observed low overlap within our human dataset, suggesting that low editing levels and RNA-Seq coverage depth might combine to explain limited overlap. Indeed, the majority of A-to-I events reside in Alu repeats and such sites are generally edited at very low levels leading to a particular lack of uniformity among samples in Alu repeats. If the comparison is not carried out at the individual A-to-I positions but at the transcript level the overlap between different databases remarkably increases, i.e. from 37% to 80% in the comparison with RADAR. The same pattern is observed at tissue level. In brain, for example, editing overlap raised from 30% to more than 70% (Supplementary Table 2). However, low overlap was always observed for muscle in which the reduced number of detected RNA editing events prevented the comparison at individual as well as transcript level (Supplementary Table 2). While a different number of A-to-I events were identified between donors, RNA editing was consistent across all three individuals with an average pairwise overlap of 80% (Supplementary Table 2).

**Figure 3:**
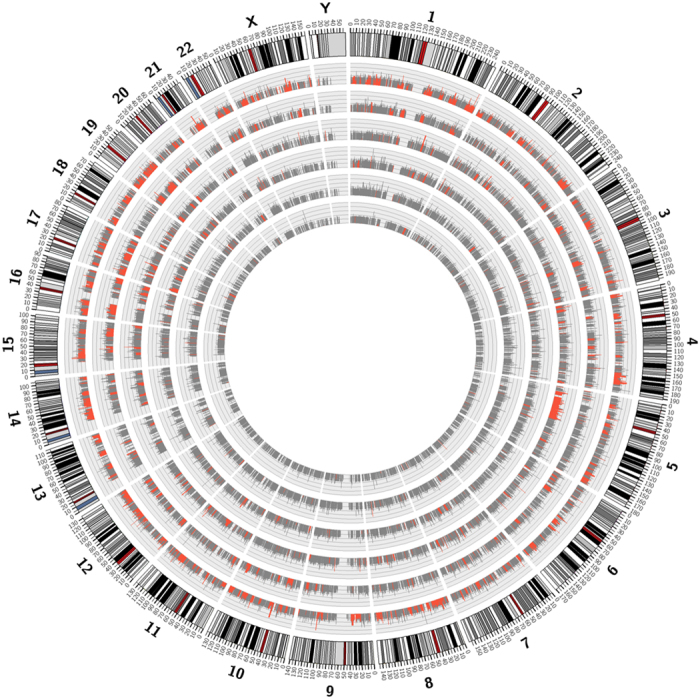
RNA editing in human transcriptomes. Whole human genome is shown as a circle in which we report for each chromosome and tissue RNA editing levels in gray bars. Tissues are shown in concentric circles and ordered as follow from the outside: BRAIN, LUNG, KIDNEY, LIVER, HEART and MUSCLE. Red bars indicate tissue specific RNA editing levels. The image was generated by Circos tool^57^.

More than 2 million of the editing events identified here are novel and our resource is the largest single collection of human editing events. Our data further highlight the pervasive nature of A-I editing (Fig. 3) and are consistent with important functional roles for editing in the modulation of gene expression dynamics.

### Characteristics of human inosinome

In agreement with previous studies in humans^9^,^12^,^16^, the vast majority edited sites (97%) reside in repetitive regions and 90% occur in Alu elements (Fig. 2B). Only 3% of edited sites occur in non-repetitive regions (Fig. 2B). The largest fraction (73%) of detected events were in introns and intergenic regions enriched in Alu repetitive elements (14%) (Fig. 2C). Ten per cent of events were in non-coding RNAs, although 88% of such sites were located in intronic regions (Fig. 2C). The large number of detected intronic sites is consistent with other studies in mammals and invertebrates^16^,^24^. It is also coherent with cotranscriptional editing previously described in Drosophila^25^.

About 2.5% of A-to-I changes were in 3′ untranslated regions (3′UTR) and several (38) of these overlapped with miRNA targets predicted by the TargetScan algorithm (Fig. 2C). Very few editing candidates (0.15%) were located in 5′ UTR regions (Fig. 2C).

Despite the large number of RNA editing events identified, only 1741 (0.05%) A-to-I changes fell in CDS regions (Fig. 2C). Of these, 903 were supported by hyper edited reads and showed low editing levels (10% on average). The remaining 838 positions were classified by ANNOVAR^26^ as synonymous or nonsynonymous. In particular, 552 events were categorized as recoding in which the top four most frequent amino acid replacements were S-to-R, K-to-R, Q-to-R and T-to-A, accounting for 40% of cases (Supplementary Figure 4). In the nonsynonymous group of sites, 401 positions reside in Alu-like elements, most likely deriving through the Alu exonization process^27^,^28^. In certain conditions, exonized Alus are included in the protein-coding region of mature transcripts (sometimes without altering the reading frame) affecting functional features of protein products and contributing to proteome variability^27^. Since Alu elements are generally edited or hyper-edited at multiple clustered positions, A-to-I changes in exonized Alus may lead to a variety of novel isoforms and protein products. RNA editing in exonized Alus may thus be required as a further mechanism to fine tune gene expression.

Nonsynonymous events in non-repetitive regions were enriched in brain (85 sites) and lung (62 sites), and included 31 out of 35 mammalian conserved recoding sites described by Pinto *et al*.^29^. Overall, we discovered 71 novel recoding events not yet included in the RADAR database^11^. These represent a very small fraction of sites, consistent with the low abundance of recoding RNA editing sites in the human transcriptome^30^.

**Figure 4:**
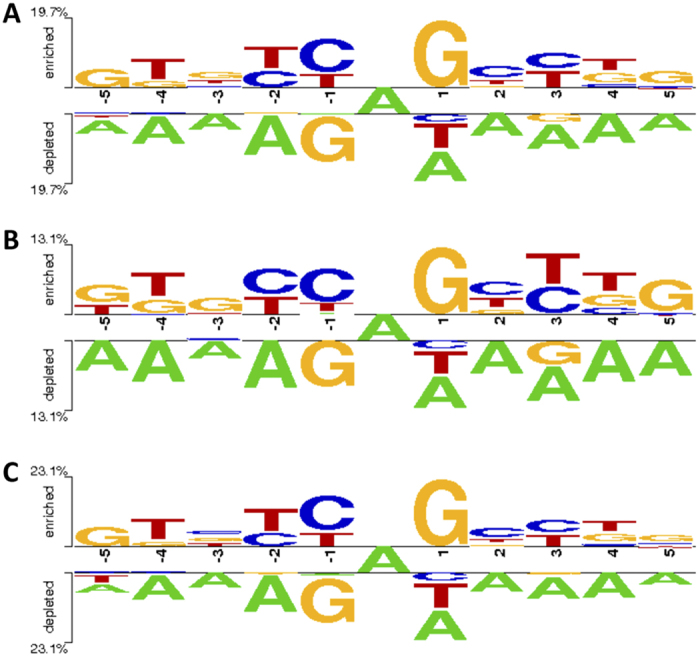
Sequence context of RNA editing sites. Sequence preferences for base positions flanking (−5, +5) detected A-to-I editing sites in (**A**) All genomic regions, (**B**) Hyper edited regions and (**C**) Non hyper edited regions. Sequence preferences were generated using the two-sample logo program^58^.

The large number of detected RNA editing sites allowed us to investigate the sequence context flanking A-to-I changes. As reported previously^31^,^32^, we observed G depletion one nucleotide downstream (−1) RNA editing sites and G enrichment one nucleotide upstream (+1) RNA editing sites (Fig. 4). Strong avoidance of G in the first nucleotide downstream (−1) editing sites was also observed in mouse^33^, *Drosophila*^24^, *Caenorhabditis*^34^ and *Acromyrmex*^35^ suggesting similar ADAR preferences for mammals and invertebrates.

Further, we noted under-representation of A in regions upstream and downstream editing sites (Fig. 4). Such depletion of A was more pronounced at the second nucleotide downstream editing positions. A similar trend was also observed in the Rhesus macaque editome^13^.

In addition, we inspected sequences flanking editing sites detected in hyper edited and non-hyper edited regions (Fig. 4). Although the nucleotide context was quite similar, we found slight sequence bias upstream hyper editing sites at positions from +2 to +5 (Fig. 4). This result would seem to suggest a distinct ADAR affinity for hyper and non-hyper edited regions.

In sequences surrounding RNA editing sites detected in Alu elements, repetitive non Alu regions and non repetitive regions (Supplementary Figs 5 and 6), we noted enrichment of C at the first position downstream (−1) of edited As in Alu repeats. The same position in repetitive non Alu regions and non repetitive regions was generally a T (Supplementary Figs 5 and 6).

Finally, we calculated the distribution of RNA editing levels and found that the median value was rather low (17%). This is due to the fact that the vast majority of A-to-I events occur in Alu repeats and typically exhibit editing levels lower than 1%^2^.

### The impact of RNA editing on human transcriptome

To investigate the impact of RNA editing on human transcriptome, we mapped all detected events on Gencode (v19) annotations and discovered that 17,140 loci over 55,496 (31%) underwent RNA editing in their exons and/or introns. Interestingly, most of the detected events (92%) occurred in protein coding genes, modifying 13062 loci out of 20173 annotated genes (65%). The remaining A-to-I events (8%) were distributed in the non-coding RNA fraction (ncRNA) (Supplementary Figure 7). Although many genes exhibited high editing rate in terms of number of events per locus, RBFOX1 appeared the most edited comprising 142 Alu elements and coding for a RNA-binding protein able to interact with the ataxin-2 which is involved in the familial form of spinocerebellar ataxia type 2 (SCA2). RBFOX1 was edited in 7699 positions, of which 6969 occurred in Alu repeats.

Since the vast majority of RNA editing events occur in Alu elements, the number of A-to-I changes per locus should be correlated with the Alu abundance per gene. In examining this relationship, we found a strong correlation of 0.78 (Pvalue = 0.0) (Supplementary Figure 8). Therefore, genes rich in embedded Alu repeats tend to be edited at higher rates.

Further, we explored the presence of RNA editing in ncRNAs and detected that a notable number (113,973) of A-to-I events resided in long-non-coding RNAs (lncRNAs), including primarily antisense RNAs and long-intergenic RNAs (lincRNAs) (Supplementary Figure 7). Two per cent of events falling in Gencode annotations occurred in known pseudogenes. Recent research has revealed several mechanisms by which pseudogenes regulate gene expression^36^,^37^. For example, pseudogenes can form double-stranded RNA by base pairing with their cognate protein-coding transcripts^37^ or compete with mRNAs for transcript stability factors^36^. In this context, RNA editing may play a functional role cooperating with pseudogenes in regulating gene expression. Indeed, A-to-I events may stabilize/destabilize higher order RNA structures or promote/prevent RNA-RNA or RNA-protein interactions.

### RNA editing in human tissues

**Figure 5:**
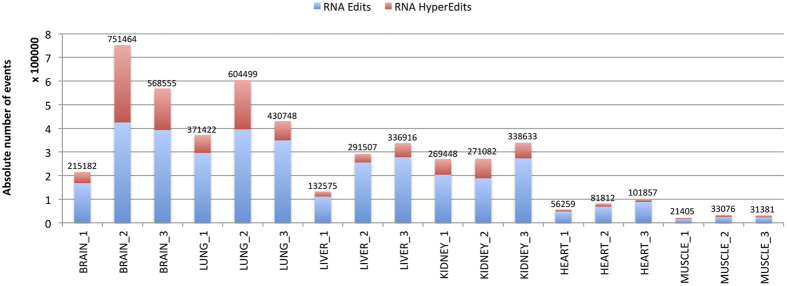
RNA editing in human tissues. Distribution of detected RNA editing events across human tissues. The absolute number of events is reported on the top of each bar. The fraction of hyper edited sites is indicated in red.

As shown in Fig. 5, the number of detected A-to-I events varied greatly among tissues and individuals (Supplementary Figure 9). This is mainly due to sequencing depth variation, stringent filters used to recover editing candidates and tissue specific roles of RNA editing. Nonetheless, we found that brain was the most edited tissue with on average 511,733 sites subjected to A-to-I change. In contrast, heart and muscle showed a smaller number of editing sites than other tissues with on average 79,976 and 28,620 modifications, respectively (Fig. 5). We detected from 30-fold to 108-fold more RNA editing candidates per tissue type (Supplementary Table 3) than a recent survey based on a limited panel of RNA-Seq data from GTEx project and a novel genome sequence-independent method for A-to-I identification^38^. The difference was remarkably evident in brain in which we detected on average 511,733 changes against 4,738. Although we produced an higher amount of RNA-Seq reads per sample, this finding suggests that the generation of a comprehensive RNA editing catalogue relies on the concomitant use of strand oriented RNA-Seq reads and whole genome sequencing from the same individual.

The total number of distinct events in brain (1,332,044) was not exceptionally high compared with other tissues as, for example, lung (1,191,711) (Supplementary Figure 10). RNA editing likely has important functional implications in non brain tissues.

**Figure 6:**
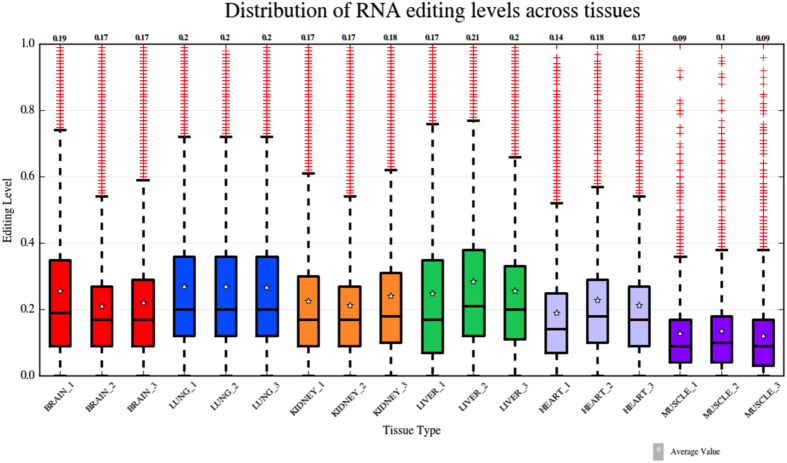
Distribution of RNA editing levels across human tissues. Boxplots showing the distributions of RNA editing levels across human tissues. Median values per sample are indicated on the top. A star inside each boxplot shows the average RNA editing level. Overall, RNA editing levels are similar across tissues and within each tissue group.

Despite the difference in the number of editing sites per sample, the distribution of RNA editing levels was quite similar across tissues and more evident within each tissue group (Fig. 6). The vast majority of events per sample showed RNA editing levels lower than 0.5 with median values ranging from 0.09 in muscle to 0.21 in liver (Fig. 6, Supplementary Figure 11). Variation coefficients, calculated as the ratio between the standard deviation and the mean RNA editing level per tissue, ranged from 0.73 in lung to 0.93 in muscle, indicating small intra-tissue variation of editing levels.

**Figure 7:**
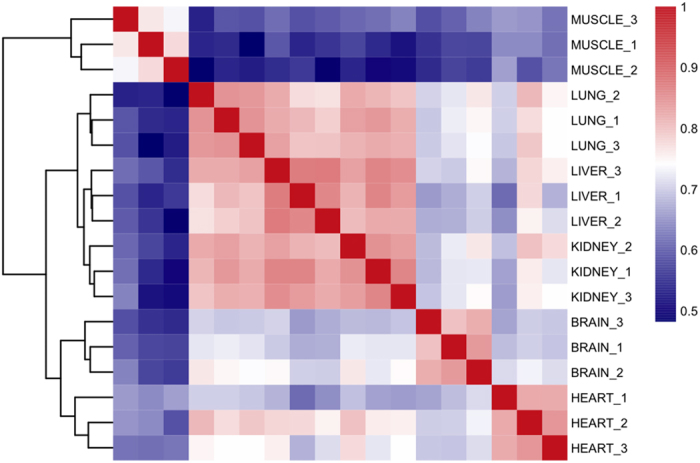
Comparison of RNA inosinomes across human tissues. The hierarchical clustering of Spearman correlation coefficients, calculated by pairwise comparisons of RNA editing levels, discriminates tissue groups and show inosinome differences across human tissues.

We also investigated the inosinome similarity across human tissues. Cluster analysis based on pairwise comparison of RNA editing levels per sample by the Spearman correlation coefficient, showed well-defined tissue segregation and three major groups involving 1) brain and heart, 2) muscle and 3) lung, liver and kidney (Fig. 7). This observation suggests that transcriptome-wide RNA editing profiles may be used to distinguish primary human tissues and may be helpful in tracing the inosinome fingerprint to characterize A-to-I events in physiological and pathological conditions. Hierarchical clustering analysis also indicates the tissue specific role of RNA editing. Notably, our results were consistent with two recent reports in human and macaque^13^,^38^, although our study included a much larger number of editing sites than previous investigations.

We also considered the genomic localization of RNA editing events across all six human tissues, finding the same trend as observed for the entire collection of sites. The largest fraction of edited positions per tissue occurred in introns and non-coding regions rich in Alu repeated elements. In addition, RNA editing events occurred mainly in protein-coding genes and the fraction of edited genes was quite similar across tissues but muscle in which only 5,564 protein coding genes out of 20,173 (27%) were modified by RNA editing.

### RNA editing in microRNAs

The main prerequisite for A-to-I RNA editing is the double-stranded RNA structure, like the one formed during the miRNA maturation process. Therefore, RNA editing is expected to affect miRNA biogenesis, through A-to-I changes in pri- or pre-miRNAs, and miRNA target interaction by A-to-I modifications in mature miRNAs, especially in the recognition site, known as the “seed” region. Previous studies focusing on RNA editing in miRNAs of human brain samples by NGS technologies have demonstrated that A-to-I changes in mature miRNAs are rare^39^,^40^. To investigate RNA editing in our human tissues, we sequenced the low molecular weight RNA fraction in each sample using the Illumina MiSeq platform. Datasets were individually uploaded to the DREAM^41^ web server in order to detect statistically significant A-to-I changes occurring in mature miRNAs. Overall, the DREAM algorithm identified 16 events, 12 of which were already known in literature (Table 2). The majority of A-to-I events occurred in brain (14) and only a few sites appeared in other tissues (Table 2 and Supplementary Figure 12). All detected positions showed low RNA editing levels, with an average value of 10%, consistent with a previous survey on miRNAs editing in the human brain^39^. Only the position chr14:101,489,681 (hg19 human genome assembly) falling in mir-411 was edited in all tissues and showed differential RNA editing levels between brain and liver, heart and muscle (P < 0.05), suggesting that mir-411 may have different functions in human tissues and that RNA editing may contribute to a further layer of regulation.

Our screen on mature miRNAs revealed 4 novel A-to-I changes, 3 of which were identified in the brain (Table 2).

In addition, we checked RNA editing changes in the full set of events detected by RNA-Seq data. Although these libraries were not prepared to capture mature miRNAs, a fraction of reads might derive from pri-miRNA transcripts and align to miRNA genes. Indeed, we found 113 A-to-I changes in genes for miRNAs, 20 of which were supported by hyper edited reads (Supplementary Table 4). Of these sites, 64 were in miRNA precursors and 49 in mature miRNAs, 7 of which were already known in the literature. Overall, most of sites (70%) occurred in brain and only two in muscle (Supplementary Figure 12). Interestingly, two positions residing in mir-1304 were consistently edited in all tissues (Supplementary Figure 13).

Finally, we noted that about 50% of detected sites (53) have been already identified in past genome wide screenings and included in the RADAR database^11^.

### ADARs expression and RNA editing profiles

**Figure 8:**
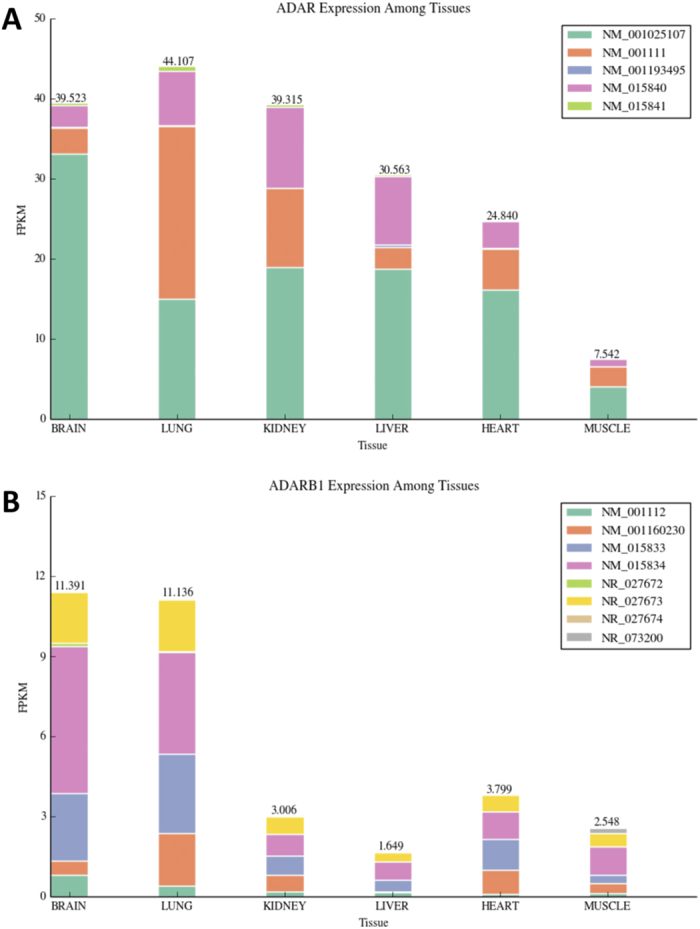
ADAR and ADARB1 expression across human tissues. Using RNA-Seq data we calculated expression values of ADAR (**A**) and ADARB1 (**B**) genes across tissues. The relative expression of known isoforms per gene locus is also reported in color.

Hierarchical clustering analysis as well as the uneven distribution of A-to-I changes across tissues indicates that RNA editing profiles are strongly tissue dependent. This behaviour may be mainly due to tissue specific regulation of ADAR enzymes and only partially to variable RNA-Seq coverage among samples. To investigate the correspondence between tissue expression profile of ADARs and RNA editing, we initially calculated expression values of ADAR genes from RNA-Seq data. As shown in Fig. 8, ADAR and ADARB1 expression decreased from brain and lung to muscle. This trend was also confirmed using independent RNA-Seq experiments from GTEx and HPA projects, in which we found correlation values of 0.93 and 0.96 for ADAR and 0.80 and 0.84 for ADARB1, respectively.

In the ADAR locus, we noted high expression level of isoform NM_001025107, coding for the short and almost exclusively nuclear p110 ADAR protein, in brain and high expression level of isoform NM_001111, coding for the interferon (IFN)-inducible p150 variant, in lung (Fig. 8). This tissue specific isoform switch may be needed to perform precise physiological requirements. In lung, for example, the IFN-inducible p150 ADAR isoform may be produced to protect against respiratory infections. Indeed, ADAR-150 has elevated deamination activity during infections and may play a role in antiviral defence against viruses that replicate in the cytoplasm^42^.

Expression values of ADARs were thus strongly correlated to inosinome profiles across tissues as both ADAR expression and RNA editing extent decreased from brain and lung to muscle. Comparing the global level of editing per sample, by summing editing levels over all positions, with the expression of ADARs (excluding ADARB2 since expressed only in brain as shown in Supplementary Figure 14), we found a positive and statistically significant correlation (Spearman Rho = 0.69 and P-value = 0.001 for ADAR; Spearman Rho = 0.47 and P-value = 0.05 for ADARB1) (Supplementary Figure 15). Spearman rank correlation coefficients increased to 0.72 for ADAR and 0.54 for ADARB1 when the number of editing events per sample was taken into account (Supplementary Figure 15). Repeating the comparison at tissue group level, we found a very strong correlation between both the number of editing events and the global level of editing with the expression of ADAR (Spearman Rho = 0.94 P-value = 0.005 and Spearman Rho = 0.88 P-value = 0.02, respectively) (Supplementary Figure 16). Strong correlations were also observed for ADARB1 but P-values were not statistically significant because of the relatively small number of tissue groups (n = 6) (Supplementary Figure 16). Our observations were consistent with those showed in a previous study on the macaque editome^13^.

**Figure 9:**
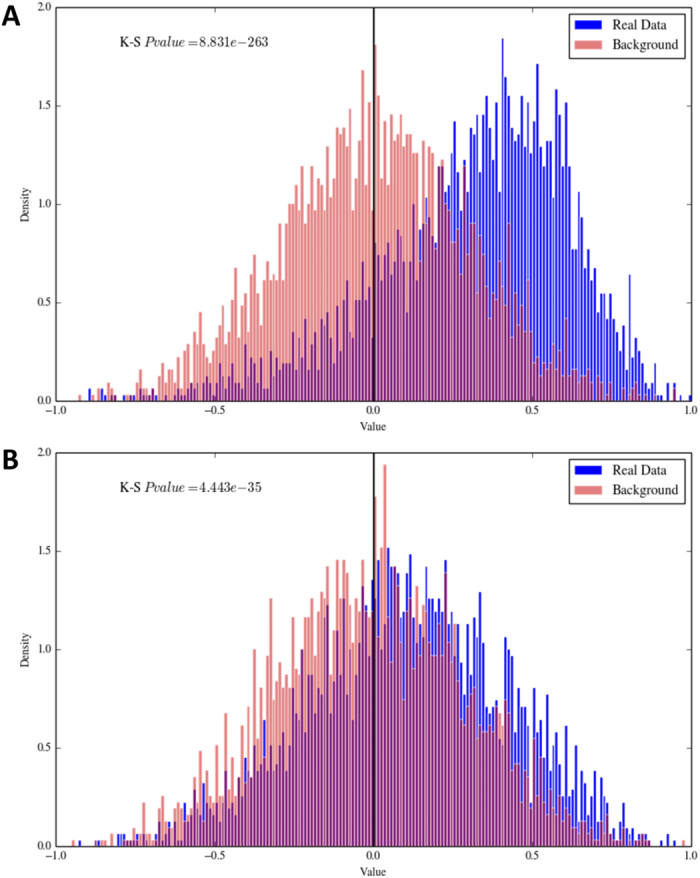
Distribution of correlation values between the expression of ADAR (in **A**) and ADARB1 (in **B**) and editing levels per positions covered by at least 10 RNA reads. K-S Pvalue is the Kolmogorov-Smirnov Pvalue.

Further, we calculated the distribution of correlation values between the expression of ADARs (ADAR and ADARB1) and editing levels per position. As background distribution we used the same dataset in which editing levels were randomly shuffled. Limiting the analysis to sites covered by at least 10 RNA reads, we found a striking and statistically significant positive correlation between ADAR expression and individual editing levels (Kolmogorov-Smirnov Pvalue = 8.83*10^−263^ against the shuffled distribution) (Fig. 9). The correlation was less for ADARB1 although still statistically significant (Kolmogorov-Smirnov Pvalue = 4.44*10^−35^ against the shuffled distribution) (Fig. 9). Together, these results support the idea that inosinome variation in human tissues strongly depends on the activity of ADARs and ADAR, rather than ADARB1, is responsible for RNA editing in repeated elements, accounting for 90% of all events^43^. Our findings fail to recover a relationship between the activity of ADARs and gene expression. Contrary to possible expectations, expression levels of edited genes are not correlated with RNA editing levels (Supplementary Figure 17) and, thus, the editing rate per gene should depend only on the expression and regulation of ADARs.

### Tissue specificity of RNA editing

As aforementioned, RNA editing profiles are strongly tissue dependent and previous studies have shown that some A-to-I events are tissue specific^31^. However, this can be due to either tissue-specific expression of target transcripts or to tissue-specific activity of ADAR enzymes (see the following paragraph). Tissue specificity of editing in humans has been rather neglected because of the absence of very large collections of editing sites and adequate datasets. We approached this issue by first selecting all genes with tissue specific expression, according to FPKM values detected by RNA-Seq in each tissue. We considered as expressed all genes showing a FPKM higher than 1. Then, we mapped the entire collection of detected RNA editing sites on expressed and tissue specific genes, excluding positions with editing evidence in more than one tissue. This last step was introduced to remove A-to-I changes occurring in very low expressed genes with FPKM values under our prefixed cut-off.

Interestingly, we found that brain contained the highest number of tissue specific RNA editing events (82,288), 5-fold more sites than in kidney (14,591) and 620-fold more sites than in muscle (Supplementary Table 5). This result is consistent with the view that RNA editing plays essential functional roles in brain. Brain also showed the highest proportion of edited tissue specific genes (45,7%) (Supplementary Table 5).

In addition, we investigated the enrichment of disease-associated genes in each list of tissue specific edited genes. Remarkably, we discovered significant enrichments for diseases typical of the tissue under investigation. For instance, brain specific edited genes were enriched in neurological and neurodegenerative disorders while kidney specific edited genes were enriched in urogenital diseases (Supplementary Table 6). Together, these findings corroborate the relevant biological role of RNA editing underlining that its deregulation in key tissue specific edited genes may lead to a variety of human disorders.

### Differential RNA editing

Differential editing levels of individual sites has been proposed to underlie deregulated A-to-I sites linked to human diseases. Differential editing has been addressed only for a handful of recoding sites, since they are generally considered more informative than editing events residing in other genomic regions and more likely associated to pathological phenotypes. Here, we investigated differential RNA editing across human tissues using RNA-seq data and performing tissue pairwise comparisons of detected events. Since the RNA-Seq coverage is not uniform among independent samples, we selected only positions showing editing evidence in all samples of each tissue group and supported by more than 10 RNA reads. A-to-I changes displaying statistically significant differential editing, after the paired t-test with P-value corrected by Benjamini–Hochberg procedure, were recovered.

Overall, we detected 2,636 sites with significant differential editing level across all six tissues (Supplementary Table 7). Such sites may also have relevant functional roles in physiological conditions. We also identified positions in which differential editing levels showed an opposite trend than differential gene expression. For example, the position chr4:57,976,234 corresponding to the recoding K-R site in the IGFBP7 gene exhibited differential editing between kidney and muscle with average editing levels of 37% and 65%, respectively, whereas IGFBP7 expression levels were much higher in kidney (796.4 FPKM) than in muscle (81.7 FPKM) (Supplementary Table 7).

These findings suggest an active functional role of RNA editing and corroborate our previous evidence of lack of correlation between gene expression and RNA editing levels (Supplementary Figure 17). We found also clusters of A-to-I changes in Alu repeats with differential editing levels across tissues but without differential expression of target genes (Supplementary Table 7). These positions deserve particular attention since may be involved in the tissue specific regulation of gene expression, especially when located in untranslated regions of mRNAs.

### The Human RNA editing atlas

Our work reports the largest collection of RNA editing events in human tissues comprising more than 3 millions of sites. To make available our entire collection as an atlas of RNA editing in human, we loaded all detected A-to-I events in a SQL database. The RNA editing atlas, accessible at http://srv00.ibbe.cnr.it/editing/, is freely available and can be interrogated by genomic region, gene name and other features as well as the tissue of origin. Query results are shown in sortable and downloadable tables in which the main characteristics of individual RNA editing events are reported. Unlike other collections, we provide RNA-Seq and DNA-Seq coverage per site as well as the RNA editing level.

## Discussion

High throughput sequencing has dramatically improved our appreciation of complex eukaryotic transcriptomes providing an opportunity to investigate co- and post-transcriptional mechanisms such as alternative splicing and RNA editing at single nucleotide resolution^9^,^44^. In human, the concomitant analysis of RNA-Seq and DNA-Seq (whole genome) from the same individual has revealed the pervasive nature of RNA editing, mostly due to the deamination of adenosine to inosine by the ADAR family of enzymes^9^. Although hundreds of thousands RNA editing events have been uncovered in human up to now, the detection of A-to-I changes from high throughput sequencing data remains computationally challenging. Sequencing errors as well as mismapping errors and single nucleotide polymorphisms prevent the compilation of an exhaustive catalogue of RNA editing in human and other organisms. Approaches working on RNA-Seq data alone have been released^16^,^38^ but they yield a limited number of sites per sample and, thus, provide low-resolution snapshots of RNA editing. In addition, the majority of computational methods to identify RNA editing in high throughput sequencing data has been applied to cell lines such as lymphoblastoid lines, which do not represent the elective material for RNA editing investigations. Only a handful of datasets comprising RNA-Seq and DNA-Seq from the same individual are publicly available and these are not derived from primary human tissues. The recent project GTEx has generated a huge amount of RNA-Seq data in human in order to uncover unknown aspects of gene expression across tissues and in a large cohort of individuals^44^,^45^. At the moment, however, GTEx does not include whole genome sequencing data and, thus, available RNA-Seq experiments may provide only a limited overview of the impact of RNA editing in human primary tissues.

RNA editing by A-to-I deamination may be extremely relevant for modulating gene expression in human transcriptomes^43^. Indeed, its deregulation has been linked to a variety of diseases including neurological/neurodegenerative disorders and cancer^5^,^46^. A comprehensive catalogue of RNA editing events in human primary tissues is a basic prerequisite for an understanding of the importance of RNA editing and its impact on cellular homeostasis in humans.

Here we produced high throughput strand specific RNA-Seq data from six tissues (brain, lung, kidney, liver, heart and muscle) from three post-mortem healthy individuals. To accurately call RNA editing events, we resequenced the whole exome and genome of each individual (Supplementary Table 1). All data were analysed with an improved computational strategy including a recent approach to detect hyper-edited reads^18^. Overall, we discovered 3,041,422 events, allowing us the characterization of the largest RNA editing collection in human with more than 2 millions of novel positions. More than 98% of detected DNA-RNA changes were of A-to-G type, meaning that RNA editing by adenosine deamination is conspicuous in human transcriptomes. Unlike past computational methods to identify RNA editing events, we introduced several algorithmic improvements leading to a higher A-to-G rate in nonsynonymous positions in which the prediction is quite challenging. We found that the majority of A-to-I changes resided in Alu repeats since they are prone to form double-stranded structures mostly inside intronic regions. On the contrary, editing events in protein coding regions were rare.

Comparing RNA editing profiles across human tissues we discovered an uneven distribution of the number of events, even in the same tissue group, that is mainly due to differences in the RNA-Seq coverage. Despite this observation, some tissues such as brain and lung appeared more edited that others like heart and muscle. In particular, muscle was the tissue with the lowest number of A-to-I changes. Nonetheless, we notably found that RNA editing profiles clustered according to tissue type. This result is particularly relevant for investigations in pathological conditions. Indeed, the direct comparison of inosinomes in different conditions allows the identification of potential anomalies at RNA editing level or the discovery of global editing dysregulation.

The clustering of whole editing profiles highlights the tissue specific regulation of RNA editing. We noted that gene expression of ADAR enzymes was strongly correlated with the number of events and the global RNA editing level per tissue. Notably, we showed that ADAR expression was more strictly correlated with editing levels than ADARB1 expression and this finding is perfectly consistent with a recent genomic analysis of ADAR binding in which the majority of CLIP-Seq sites were located in Alu repeats^47^, confirming ADAR as the main responsible of observed A-to-I changes.

Our results, therefore, suggest that tissue-specific inosinomes depend on the expression of ADARs and are regulated by specific tissue requests. In addition, RNA editing levels are not associated to gene expression meaning that the increased expression of a given gene does not entail higher editing levels.

The pervasive RNA editing landscape in the human transcriptome raises essential and intriguing questions about functional roles of A-to-I changes. Experiments involving ADAR enzymes knock-out in different model organisms have clearly demonstrated the indispensability of RNA editing in survival and in preserving the cellular homeostasis^7^,^8^. In human, ADAR dysfunction or altered editing levels have been linked to a variety of disorders affecting mainly the nervous central system^5^,^46^.

Our genome wide screening indicates that more than 90% of RNA editing sites resides in known protein coding genes (affecting about 65% of mRNAs) and may profoundly affect transcriptome dynamics with a variety of functional consequences. This makes RNA editing an essential and vital mechanism in human. Employing our large collection of A-to-I modifications, we investigated the indispensability of RNA editing in human, exploring the relationship between inosinome and human diseases. To this end, we downloaded all known genes associated to human disorders from DisGeNET database comprising over 380,000 associations between more than 16,000 genes and 13,000 diseases^48^. Next, we calculated the enrichment in our set of 13,062 edited protein-coding genes, if any. Surprisingly, we found that edited genes were consistently enriched in genes involved in neurological disorders and cancer (Supplementary Table 8). We confirmed these results using also the web service DAVID^49^ to search into the genetic association database^50^ (Supplementary Table 8).

In addition, we investigated how many edited genes were in common with a collection of human essential genes obtained from DEG database^51^. Essential genes are those indispensable for cellular survival and associated with a wide spectrum of diseases affecting diverse physiological systems^52^. Our screen revealed that 74% (1842/2501) of essential genes were in the edited set, confirming once again the importance of RNA editing and its potential functional impact in the human transcriptome.

Another important aspect emerging from our results is the relationship between RNA editing and human diseases of the nervous central system. To explain this relationship we focused on tissue specific genes and RNA editing events occurring herein. The majority of edited genes are specifically expressed in brain than in other tissues, indicating that RNA editing may play indispensable functional roles in brain. However, edited genes in other tissues deserve also careful attention since they are associated to tissue specific pathologies.

In conclusion, our study provide a comprehensive overview of RNA editing in human primary tissues, highlighting inosinome variations and the importance of using matched RNA-Seq and DNA-Seq data from the same individual to accurately profile RNA editing. Our collection is freely available through the web and may be a relevant resource to investigate editing alterations in human disorders. We think that RNA editing deregulation may be a key phenomenon in many diseases and the understanding of mechanisms relating edited genes and disrupted cellular homeostasis may lead to new biomarkers and hopefully to the design of novel biotechnological drugs.

## Methods

### Samples and nucleic acids extraction

Six different post-mortem human snap-frozen tissues (brain, liver, lung, striated muscle, kidney, heart) from three unrelated “nondiseased” Caucasian individuals (males, aged 47–54) were obtained from Cureline (South San Francisco, CA, USA). Sample IDs and further details are reported in Supplementary Table 1.

DNA was extracted and purified using the DNeasy Blood & Tissue Kit (Qiagen, Hilden, Germany) according to the manufacturer’s instructions. Each sample was then quantified and qualitatively checked by the NanoDrop 2000c (Thermo Fisher Scientific, USA).

Total RNA was extracted and purified using the RNeasy Plus Mini Kit (Qiagen, Hilden, Germany), according to the manufacturer’s instructions. RNA quality was assessed by the Agilent Bioanalyzer 2100, obtaining RIN (RNA Integrity Number) values ranging from 5 to 7, that were considered acceptable for RNA derived from post-mortem tissues.

### Exome sequencing

Exome capture was performed using the TruSeq Exome Enrichment Kit (Illumina, San Diego, CA), according to the manufacturer’s instructions. Briefly, for each tissue, a DNA library, including inserts ranging in size from 200 to 400 bp approximately, was prepared using the TruSeq DNA Sample Prep kit (Illumina). Then, each library was hybridated with biotinylated probes targeting the exonic regions (about 200,000 exons, covering about 62 Mb of the human genome). After two steps of enrichment with the probes, the captured exonic regions were sequenced on the Illumina HiSeq2000 platform at IGA Technology Service in Udine (Italy). Paired-end reads of 100 bp were generated for each fragment (Supplementary Table 1).

### Strand-oriented RNA-Sequencing

For each tissue, a strand-oriented RNA library was prepared to preserve information about which DNA strand was the original template during the synthesis of transcripts, thus offering strand orientation for detection of antisense transcription and providing information about regulatory relationships.

The cytoplasmatic rRNA removal was performed for each total RNA sample using the Ribo-Zero rRNA removal Kit (Epicentre, Madison, WI, USA). The rRNA-depleted RNA was used to prepare the stranded-oriented RNA-seq library using the TruSeq Stranded Total RNA Sample Prep Kit (Illumina, San Diego, CA, USA), according to the manufacturer’s instructions. Briefly, each RNA was chemically fragmented prior to the random priming reverse transcription reaction for first strand cDNA generation. The fragmentation step resulted in a RNA-seq library including inserts ranging in size from approximately 100–400 bp. During the second strand synthesis, dUTP was incorporated in place of dTTP, thus preventing amplification of this strand during the subsequent PCR step and retaining strand information. cDNA libraries were sequenced on the Illumina HiSeq2500 platform at IGA Technology Services in Udine (Italy). Paired-end reads of 100 nt were generated for each fragment (Supplementary Table 1).

### Whole genome sequencing

Brain DNA, from same individuals for which RNA and exome sequencing was performed, was used to create three DNA libraries by the TruSeq DNA Sample Prep kit (Illumina), including inserts ranging in size from 200 to 400 bp approximately. Then, each library was sequenced on the Illumina HiSeq2000 platform at Personal Genomics Service in Verona (Italy). Paired-end reads of 100 bp were generated for each fragment (Supplementary Table 1).

### MiRNA sequencing

Indexed cDNA libraries from the RNA fraction at low molecular were prepared using the TruSeq small RNA sample Preparation kit (Illumina, San Diego, CA) according to the manufacturer’s protocol and recommendations. Single end sequencing (1 × 50 b), after fluorimetric quantification, was performed on the Illumina Miseq platform.

### Exomic and genomic read alignments

Reads from exome and whole genome sequencing were initially checked for quality by FASTQC (http://www.bioinformatics.babraham.ac.uk/projects/fastqc/) and, subsequently, trimmed at 3′ end to remove adaptor sequence contamination by TrimGalore (http://www.bioinformatics.babraham.ac.uk/projects/trim_galore/). Cleaned reads were then aligned to the GRCh37 human reference genome using GSNAP^19^. The resulting SAM files were converted to BAM files by SAMtools^53^ and deprived of PCR duplicates using Picard (http://broadinstitute.github.io/picard/). Main mapping statistics were calculated using Picard and custom python scripts (Supplementary Table 1).

### Alignment of RNA-Seq reads

RNA-Seq reads in FASTQ format were inspected using FASTQC program. Adaptors and low quality regions (phred cutoff of 20) were trimmed using TrimGalore, excluding reads with final length less than 50 bases.

Cleaned reads were subsequently aligned onto the complete GRCh37 human genome by means of GSNAP^19^ (using as parameters: -B 5 -d hg19 -t5 -s splicesites -E1000 -N1 -n1 -Q -O –nofails -A sam –force-xs-dir -a paired) providing a list of exon-exon junctions from Ensembl, UCSC and RefSeq databases. Unique and concordant alignments in SAM format were converted in the binary BAM format by SAMtools^53^ and basic statistics were calculated using Picard tools (CollectRnaSeqMetrics.jar) (Supplementary Table 1).

Transcriptome quantification and differential expression was performed using Cufflinks^54^ and CuffDiff2^55^ software. Reference human transcriptome was obtained from iGenomes repository (http://support.illumina.com/sequencing/sequencing_software/igenome.html) and annotations for rRNA genes were downloaded from UCSC genome browser selecting the RepeatMask table.

### RNA editing detection

RNA editing candidates were detected using a pipeline based on our REDItools suite^20^,^56^. In particular, we applied REDItoolDnaRna.py to each BAM file obtained by the mapping of RNA-Seq data from human tissues using GSNAP (see the “Alignment of RNA-Seq reads” paragraph above). Initially, nucleotide changes were called using loose parameters (-c 1,1 -m 20,20 -v1 -q25,25 -s2 -g2 -S -e -n0.0 -N0.0 -u -l). Then, read pairs harbouring nucleotide changes were realigned onto the human genome (hg19 assembly version) using Blat and only uniquely mapping pairs were retained. Base changes residing in not unique read alignments were discarded. In addition, we removed positions surrounding genomic regions (+/− 10 bases) in which the multiple alignment of reads was not optimal by the presence of indels.

Resulting tables were subsequently updated adding genomic and exomic information by means of the REDItoolAddGenome.py script. Individual positions in updated table were finally annotated using the AnnotateTable.py script and the following databases: RepeatMask, dbSNP (v. 138) and RetroposedGenes from UCSC. For each table, we separated positions residing in Alu elements, repetitive non-Alu regions and non-repetitive regions using custom bash scripts. During the split, we retained only positions supported by at least 10 genomic reads and completely homozygous. DNA-RNA changes in retroposed genes were eliminated as well as sites in which the alternative nucleotide was supported by less than 2 RNA reads. RNA editing candidates in repetitive non-Alu regions and non-repetitive regions underwent more stringent filters. Indeed, we excluded positions in the first and last 6 bases of reads, with quality score less than 30, with DNA-RNA frequency change lower than 0.1, in homopolymeric regions longer than 5 bases, in which the alternative nucleotide was supported by less than 3 RNA reads. In addition, we removed duplicated reads and applied again the Blat filter. Resulting tables were used for downstream analyses by means of custom scripts.

RNA editing sites in hyper-edited reads were detected using the pipeline described in Porath *et al*.^18^.

All detected positions were finally annotated by ANNOVAR^26^.

The comparison between our detected editing sites and available RNA editing databases, such as DARNED and RADAR, was performed by a custom script. DARNED annotations for human were downloaded from http://beamish.ucc.ie/. Version 2 RADAR annotations were downloaded from http://rnaedit.com/.

### The Human RNA editing atlas

A specialized database, containing the complete collection of human editing events with an interface designed to facilitate browsing and downloading of selected positions was designed in SQL using dedicated python scripts based on the sqlite3 module. Interactive tables showing results are dynamically generated using the JQuery plug-in DataTables (https://www.datatables.net/).

### RNA editing in miRNAs

RNA editing candidates in mature miRNAs were detected using the DREAM^41^ web server implementing the algorithm already described in Alon *et al*.^39^. Reads in FASTQ format were uploaded in DREAM and analysed using default parameters. The resulting textual tables were parsed using custom scripts and genomic positions corresponding to predicted RNA editing events were contrasted with DNA-Seq reads and dbSNP138 in order to remove potential SNPs.

### Gene enrichment

Enrichment in specific gene categories was calculated using custom scripts. In case of genes associated to diseases, all annotations were downloaded from DisGeNET database^48^. Essential genes were, instead, obtained from DEG database^51^. Gene enrichments were also computed using the DAVID web tool^49^.

### Data availability

All sequencing data produced in the present work have been submitted to dbGaP database under the accession phs000870.

## Additional Information

**How to cite this article**: Picardi, E. *et al*. Profiling RNA editing in human tissues: towards the inosinome Atlas. *Sci. Rep*. **5**, 14941; doi: 10.1038/srep14941 (2015).

## Supplementary Material

Supplementary Information

## Figures and Tables

**Table 1:** Summary of samples used in the present study.

Tissue ID	Tissue Type	PMI (hrs)	Age	Sex	Ethnicity	Cause of death	RNA-Seq	miRNA-Seq	WEX	WGS
brain_1	brain	3	47	Male	Caucasian	Acute coronary syndrome	✓	✓	✓	✓
brain_2	brain	1	54	Male	Caucasian	Car accident	✓	✓	✓	✓
brain_3	brain	1	48	Male	Caucasian	Traumatic asphyxia	✓	✓	✓	✓
heart_1	heart	3	47	Male	Caucasian	Acute coronary syndrome	✓	✓	✓	
heart_2	heart	1	54	Male	Caucasian	Car accident	✓	✓	✓	
heart_3	heart	1	48	Male	Caucasian	Traumatic asphyxia	✓	✓	✓	
kidney_1	kidney	3	47	Male	Caucasian	Acute coronary syndrome	✓	✓	✓	
kidney_2	kidney	1	54	Male	Caucasian	Car accident	✓	✓	✓	
kidney_3	kidney	1	48	Male	Caucasian	Traumatic asphyxia	✓	✓	✓	
liver_1	liver	3	47	Male	Caucasian	Acute coronary syndrome	✓	✓	✓	
liver_2	liver	1	54	Male	Caucasian	Car accident	✓	✓	✓	
liver_3	liver	1	48	Male	Caucasian	Traumatic asphyxia	✓	✓	✓	
lung_1	lung	3	47	Male	Caucasian	Acute coronary syndrome	✓	✓	✓	
lung_2	lung	1	54	Male	Caucasian	Car accident	✓	✓	✓	
lung_3	lung	1	48	Male	Caucasian	Traumatic asphyxia	✓	✓	✓	
muscle_1	muscle	3	47	Male	Caucasian	Acute coronary syndrome	✓	✓	✓	
muscle_2	muscle	1	54	Male	Caucasian	Car accident	✓	✓	✓	
muscle_3	muscle	1	48	Male	Caucasian	Traumatic asphyxia	✓	✓	✓	

Omic analyses that have been performed are indicated in the last four columns. Additional details and statistics are in Supplementary Table 1. (PMI: Post Mortem Interval).

**Table 2:** List of detected RNA editing events in mature miRNAs.

Position	Name	In-pre	In-mat	known	BRAIN	LUNG	LIVER	KIDNEY	HEART	MUSCLE
chr1:1102544	hsa-mir-200b	61	5	✓				✓		
chr1:220373944	hsa-mir-664a	18	8		✓					
chr11:57408726	hsa-mir-130a	56	2	✓	✓					
chr11:59976571	hsa-mir-6503	59	7				✓		✓	
chr14:101489681	hsa-mir-411	20	5	✓	✓	✓	✓	✓	✓	✓
chr14:101507127	hsa-mir-376a-1	9	3	✓	✓					
chr14:101512308	hsa-mir-381	52	4	✓	✓		✓	✓	✓	
chr14:101513675	hsa-mir-539	18	10	✓	✓					
chr14:101514299	hsa-mir-889	62	14		✓					
chr19:52195911	hsa-mir-99b	47	3	✓	✓					
chr2:25551539	hsa-mir-1301	52	5		✓					
chr21:17911421	hsa-mir-99a	13	1	✓	✓	✓				
chr7:5535483	hsa-mir-589	66	6	✓	✓	✓				
chr8:141742704	hsa-mir-151a	49	3	✓	✓	✓				
chr9:97847790	hsa-mir-27b	64	4	✓	✓	✓				
chr9:116971745	hsa-mir-455	32	17	✓	✓	✓				

For each A-to-I change we report the name of the miRNA, the location inside the pre-miRNA (In-pre) and mature miRNA (In-mat), a flag indicating if the event has already been observed (known) and a flag indicating the target tissue.
