# Corrigendum: Profiling RNA editing in human tissues: towards the inosinome Atlas

**DOI:** 10.1038/srep20755

**Published:** 2016-02-08

**Authors:** Ernesto Picardi, Caterina Manzari, Francesca Mastropasqua, Italia Aiello, Anna Maria D’Erchia, Graziano Pesole

Scientific Reports 5: Article number: 1494110.1038/srep14941; published online: 10092015; updated: 02082016.

The original version of this Article contained typographical errors in the inosinome database link ‘ http://srv00.recas.ba.infn.it/editing’ which was incorrectly given as ‘ http://srv00.ibbe.cnr.it/editing/’. These errors have now been corrected in the PDF and HTML versions of the Article.

