# Supplementary material for: Profiling RNA editing in human tissues: towards the inosinome Atlas
Source: Sci Rep. 2015 Oct 9;5:14941. doi: 10.1038/srep14941 (PMC4598827; doi:10.1038/srep14941)
Supplement: Supplementary Information [file srep14941-s1.pdf]

Profiling RNA editing in human tissues: towards the inosinome Atlas

Ernesto Picardi[1,2,3], Caterina Manzari[2], Francesca Mastropasqua[1], Italia Aiello[1], Anna Maria D'Erchia[1,2] and Graziano Pesole[1,2,3,4*]

[1] Department of Biosciences, Biotechnology and Biopharmaceutics, University of Bari, Via Orabona 4, 70126 Bari, Italy

[2] Institute of Biomembranes and Bioenergetics, National Research Council, Via Amendola 165/A, 70126 Bari, Italy

[3] National Institute of Biostructures and Biosystems (INBB), Viale Medaglie D'Oro 305, 00136 Rome, Italy

[4] Center of Excellence in Comparative Genomics, University of Bari, Piazza Umberto I, 70121 Bari, Italy

Supplementary Figures

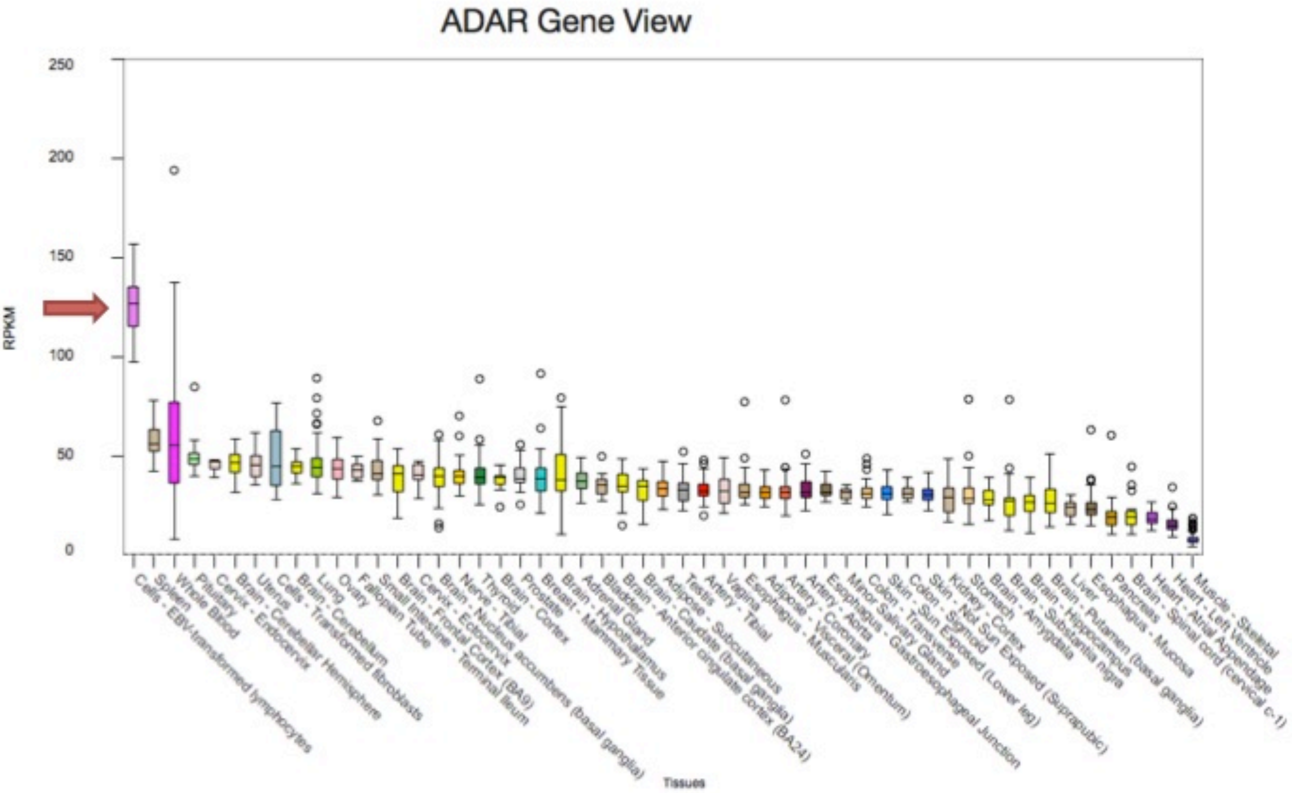

Supplementary Figure 1. ADAR expression across GTEx tissues. Lymphoblastoid cell line is indicated by the red arrow. The plot was generated at the GTEx web portal (http://www.gtexportal.org/home/).

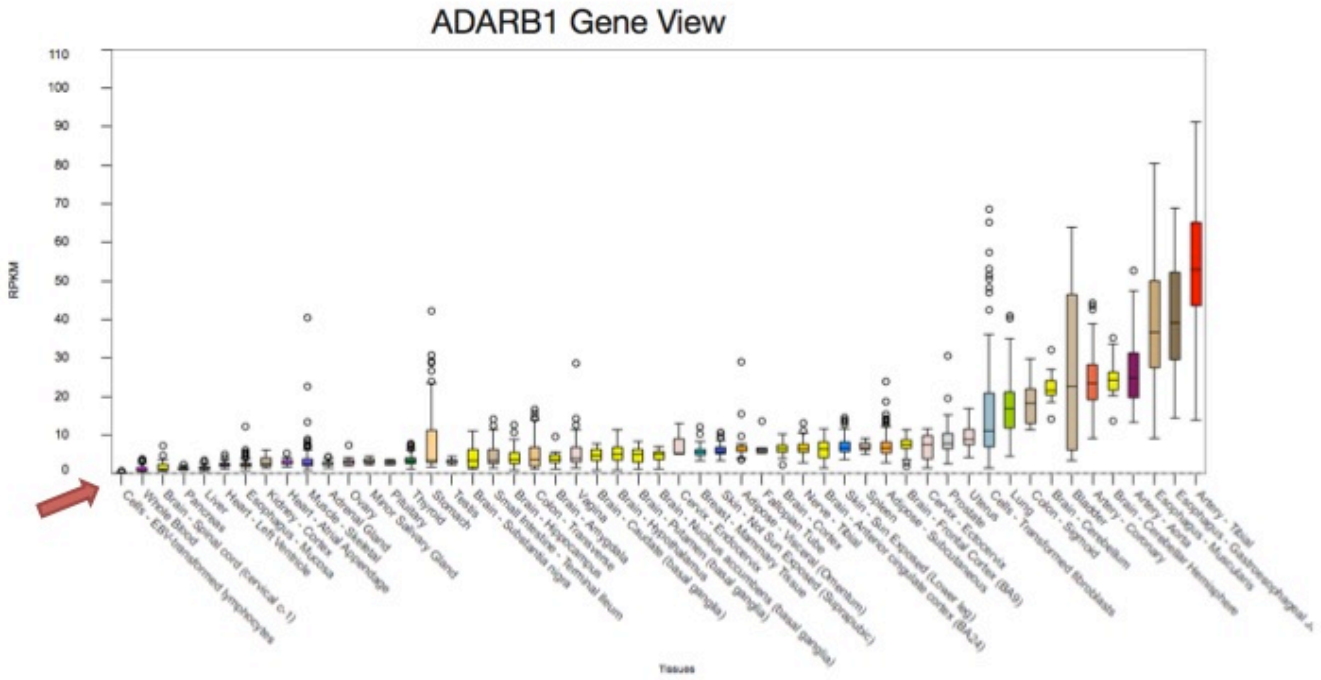

Supplementary Figure 2. ADARB1 expression across GTEx tissues. Lymphoblastoid cell line is indicated by the red arrow. The plot was generated at the GTEx web portal (http://www.gtexportal.org/home/).

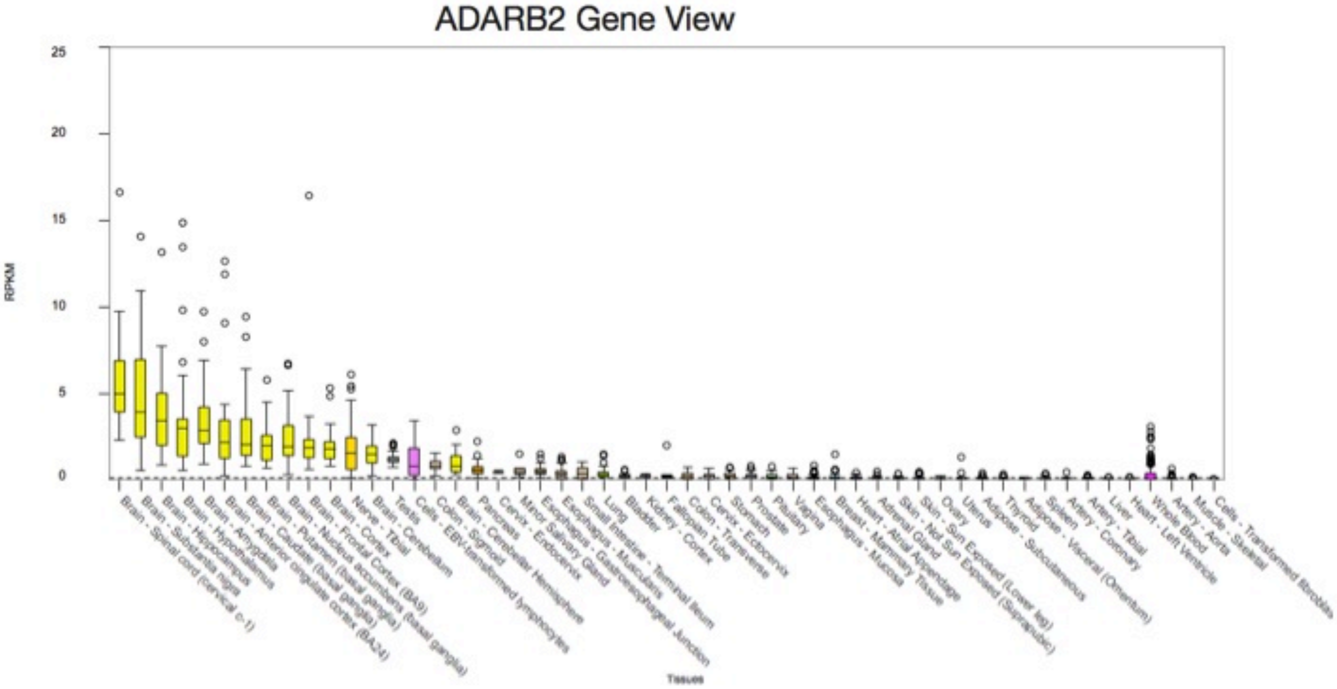

Supplementary Figure 3. ADARB2 expression across GTEx tissues. Brain tissues are in yellow. The plot was generated at the GTEx web portal (http://www.gtexportal.org/home/).

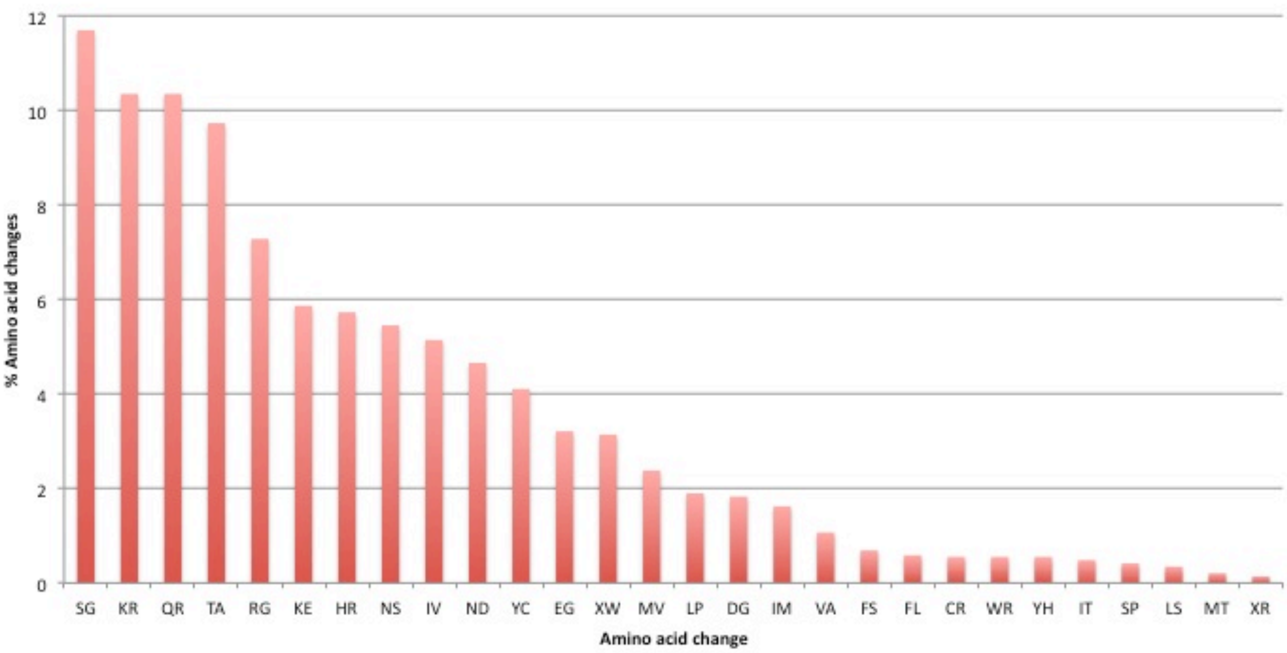

Supplementary Figure 4. Distribution of amino acid changes in recoding sites.

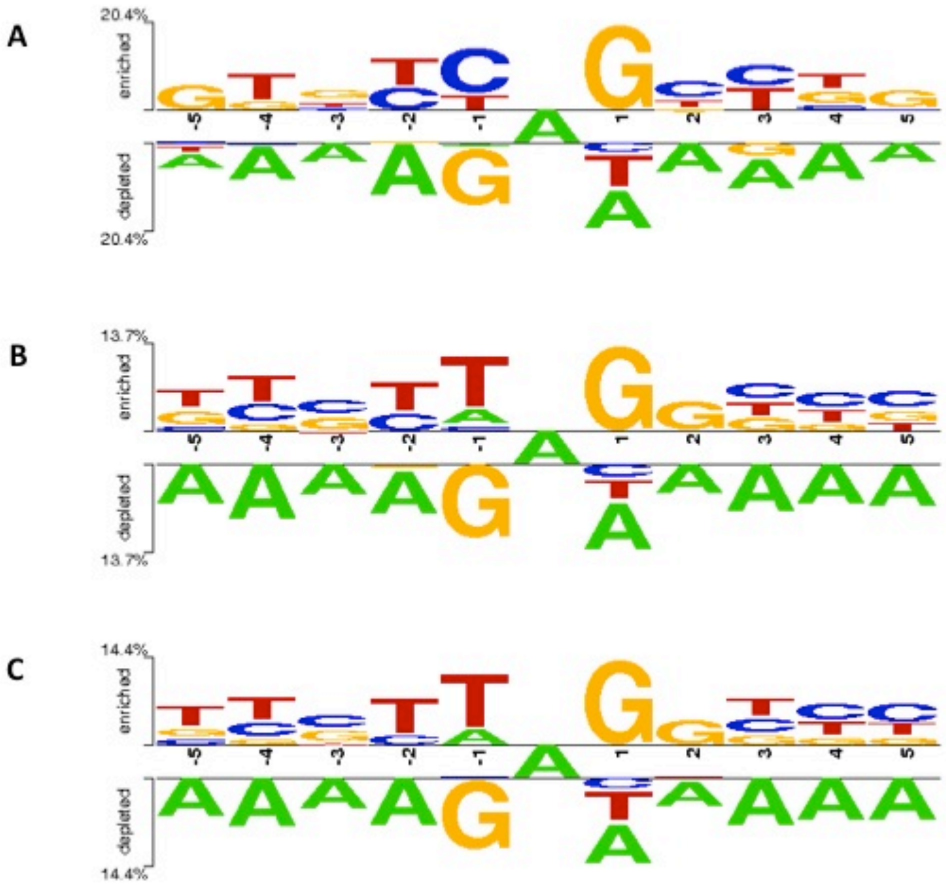

Supplementary Figure 5. Sequence context of RNA editing sites.
Sequence preferences for base positions flanking detected A-to-I editing sites in A) ALU elements, B) Repetitive non ALU regions and C) Non repetitive regions. Sequence preferences were generated using the two-sample logo program.

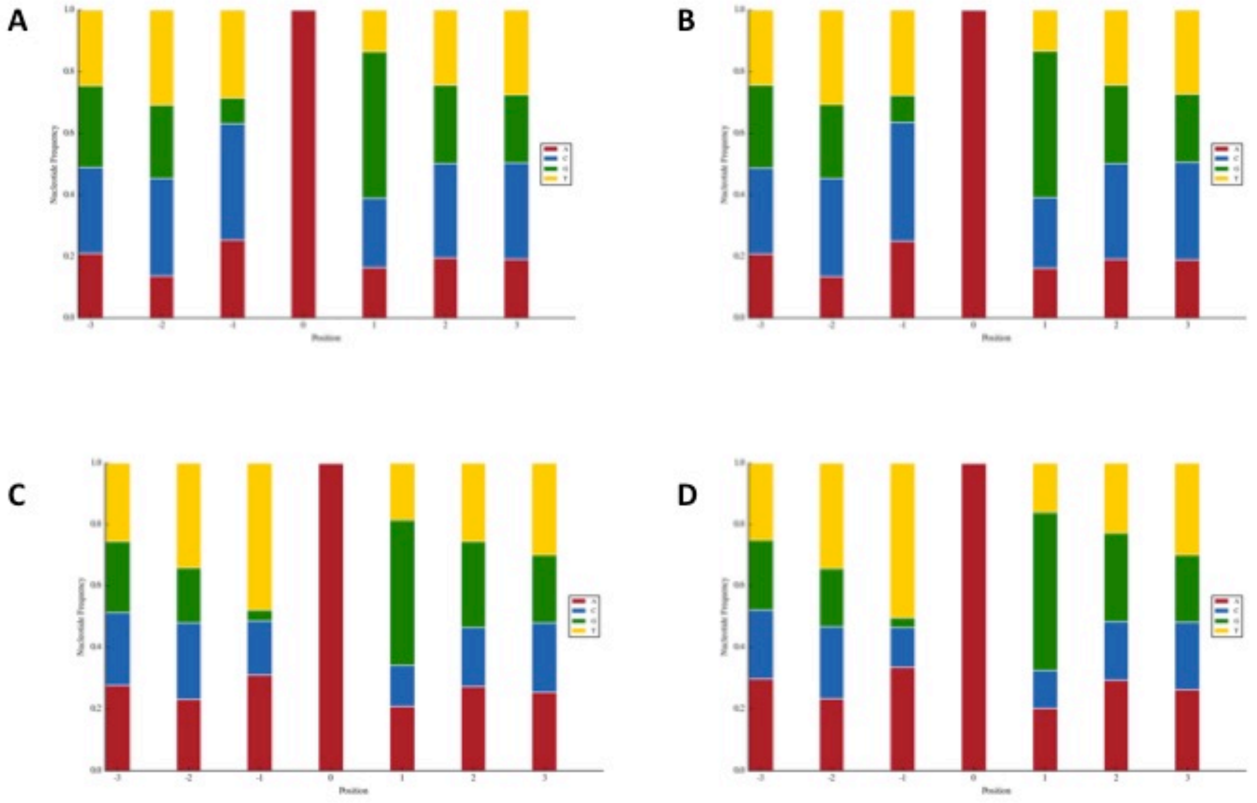

Supplementary Figure 6. Base distribution near RNA editing sites.
Base distribution near (-3,+3) editing sites calculated in: A) ALL genomic regions; B) ALU elements; C) Repetitive non ALU regions; D) Nonrepetitive regions.

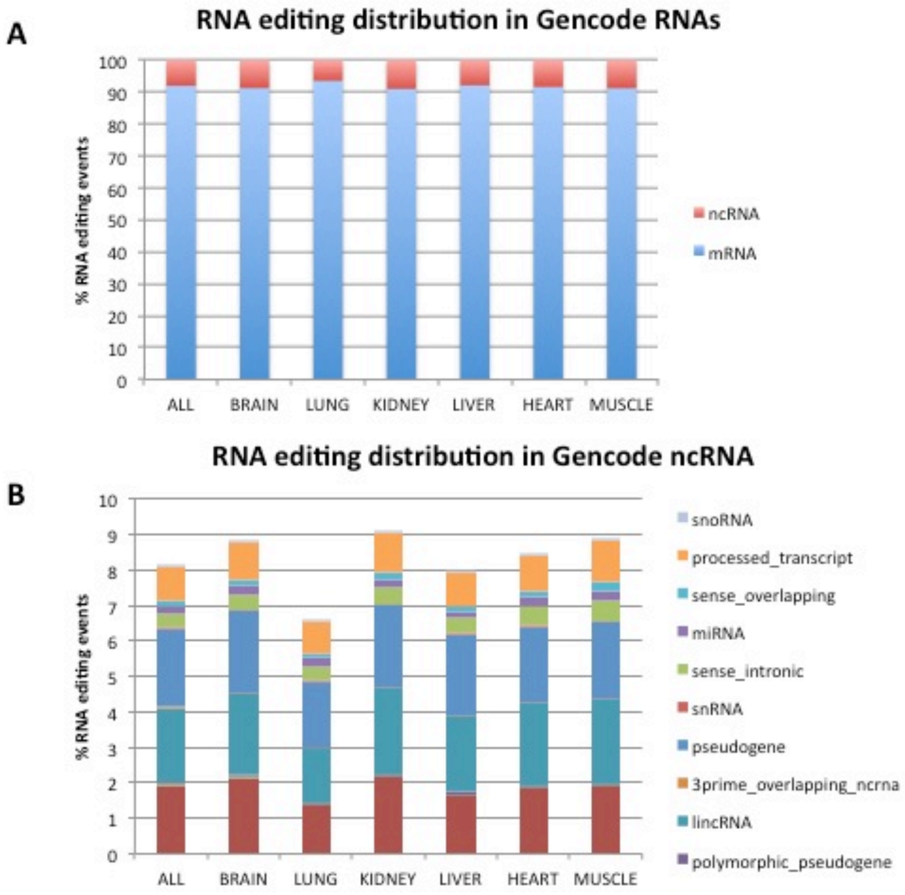

Supplementary Figure 7. RNA editing distribution in Gencode annotation. In A) we report the distribution of RNA editing events in Gencode RNAs. The vast majority of A-to-I changes resides in mRNAs. In B) we show the distribution of RNA editing events in the ncRNA fraction.

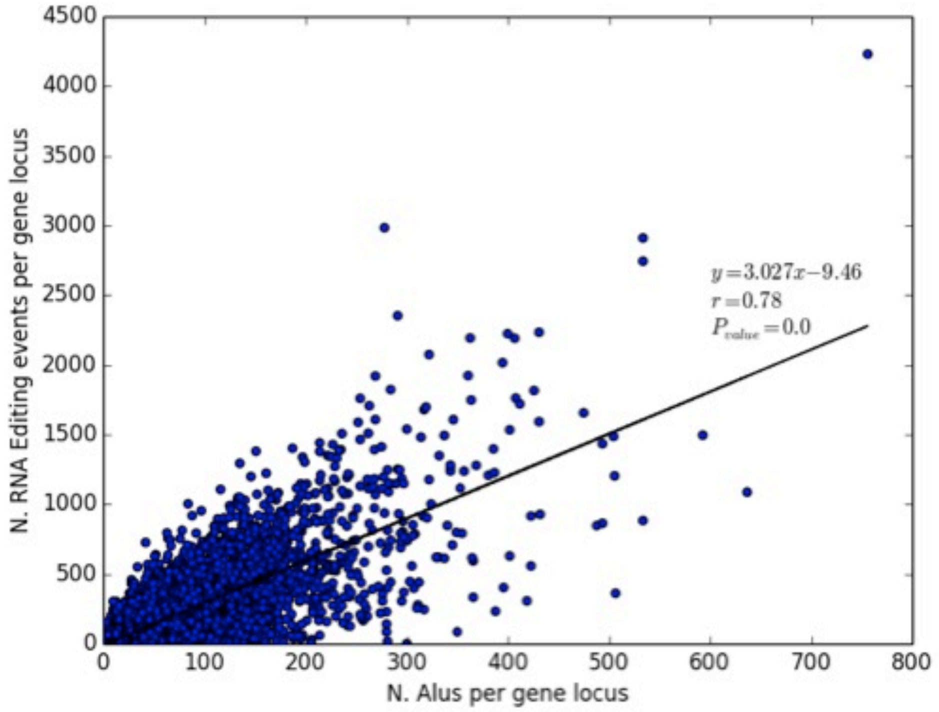

Supplementary Figure 8. Relationship between number of Alus and number of events per gene locus. We calculated the correlation between the number of Alu repeats and the number of RNA editing events per gene, finding a strong positive value of 0.78.

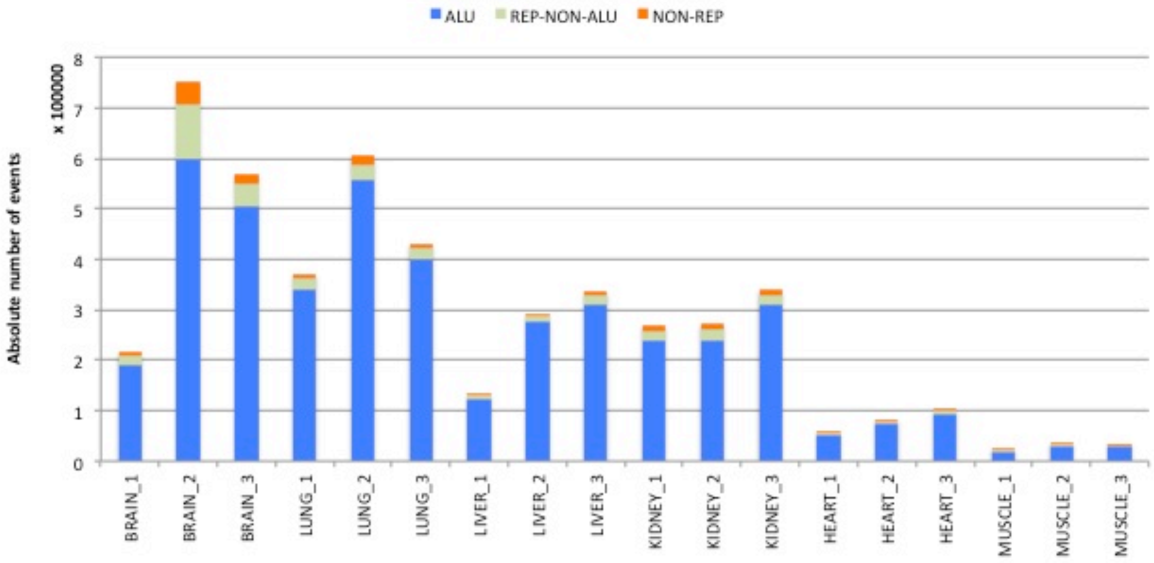

Supplementary Figure 9. RNA editing across human tissues.
Distribution of detected RNA editing events across human tissues. For each sample, we show in color the fraction of events in: ALU elements (blue), repetitive non-ALU regions (green) and non repetitive regions (red).

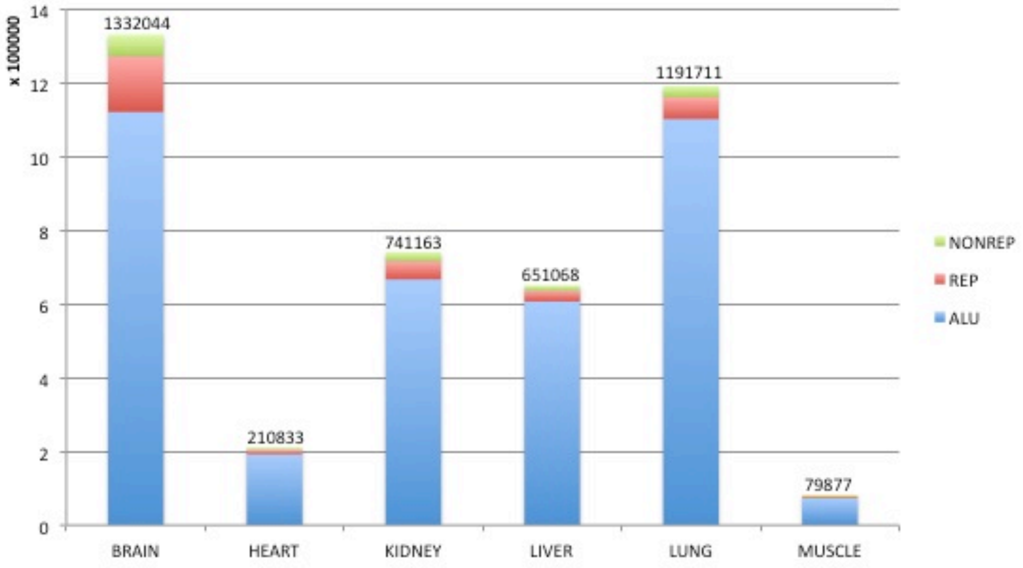

Supplementary Figure 10. The number of RNA editing events across human tissues.
The number of detected RNA editing events per tissue group. For each tissue, we show the number of events pooled from all individual and, in color, the fraction of changes in: ALU elements (blue), repetitive non-ALU regions (green) and non repetitive regions (red).

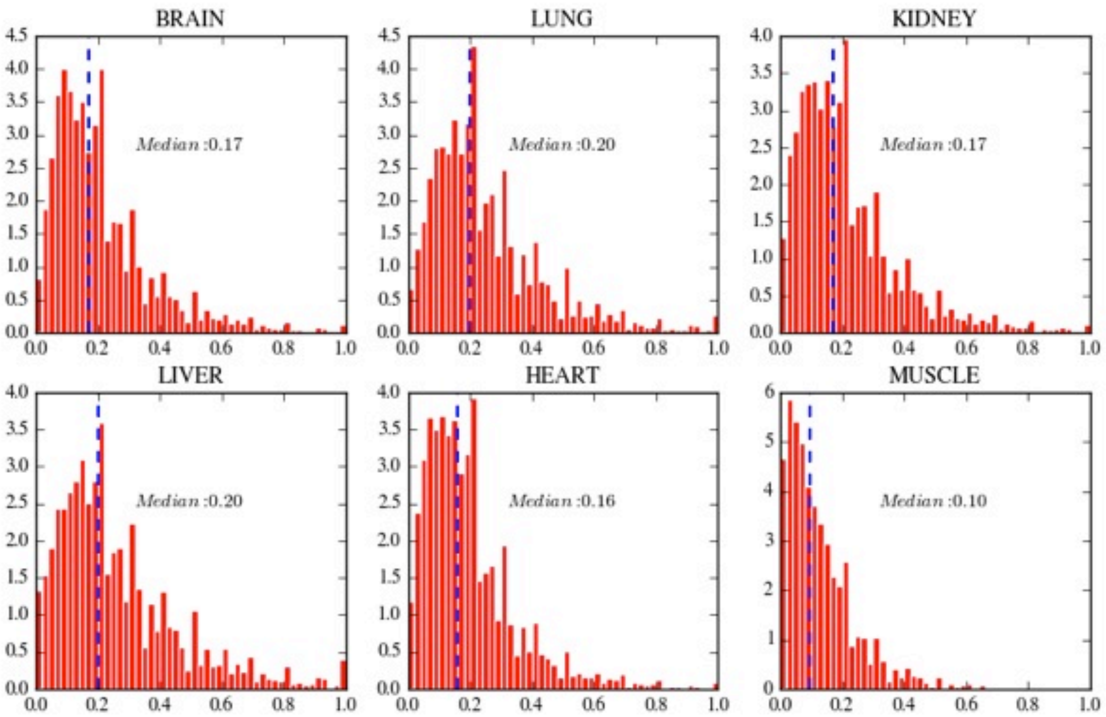

Supplementary Figure 11. Distributions of RNA editing levels across tissue groups.
Here we report the distributions of RNA editing levels across tissue groups indicating the median values. In each graph, RNA editing levels are along x-axes.

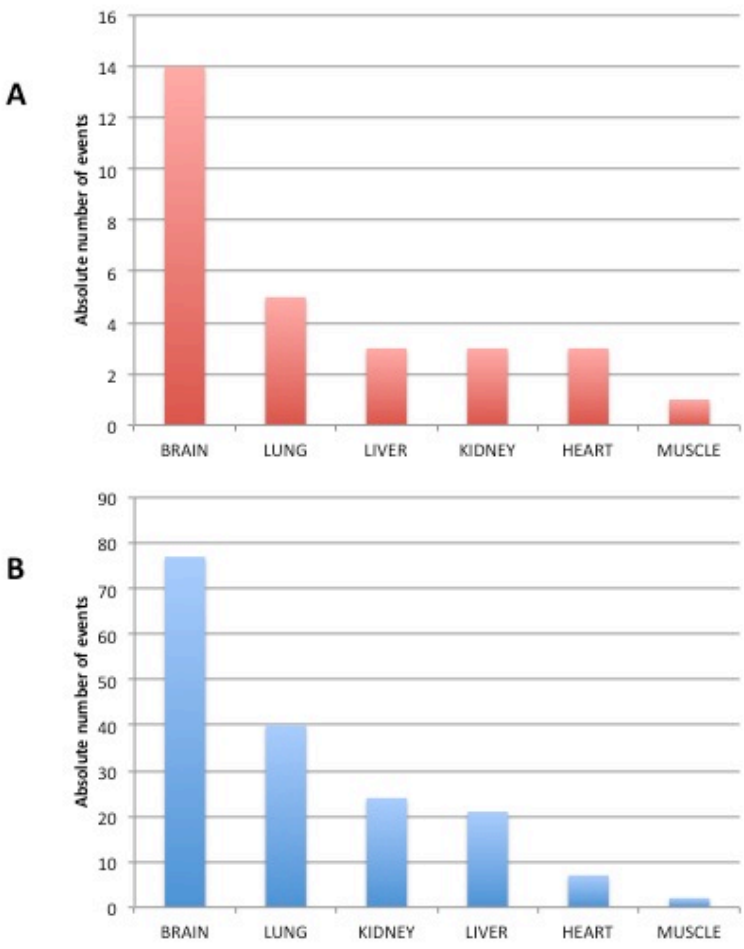

Supplementary Figure 12. RNA editing events in miRNAs across tissues as detected by miRNA-Seq (A) and RNA-Seq (B).

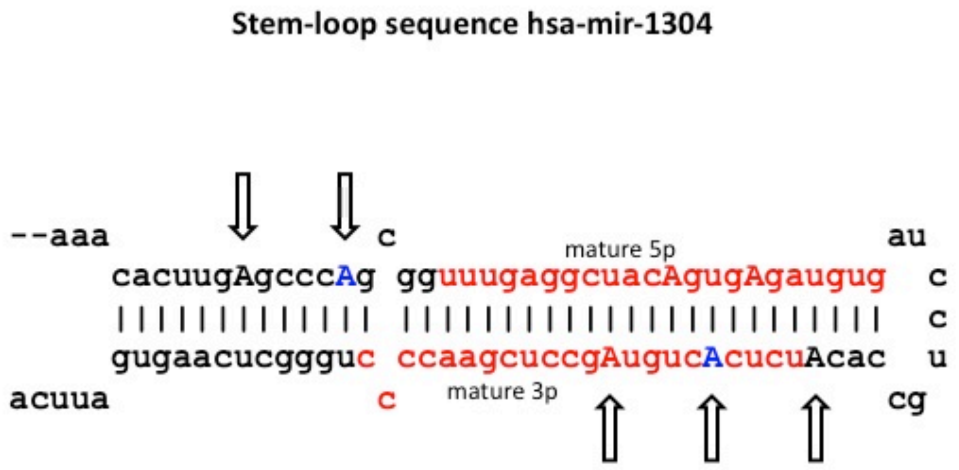

Supplementary Figure 13. Stem-loop sequence of has-mir-1304. Mature sequences are indicated in read whereas edited adenosines are in upper case. Edited residues found in all samples are colored in blue.

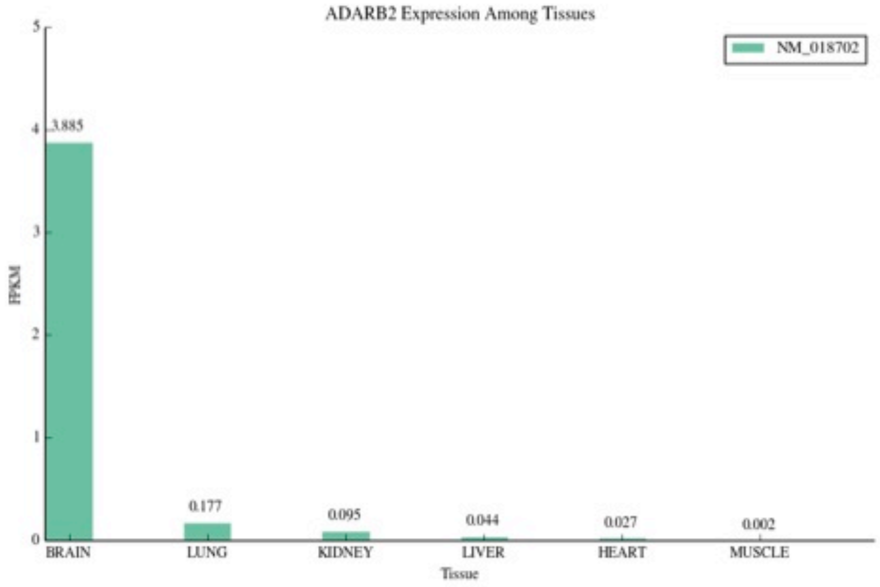

Supplementary Figure 14. ADARB2 expression across human tissues.
Expression values of ADARB2 gene across human tissues calculated by RNA-Seq data.

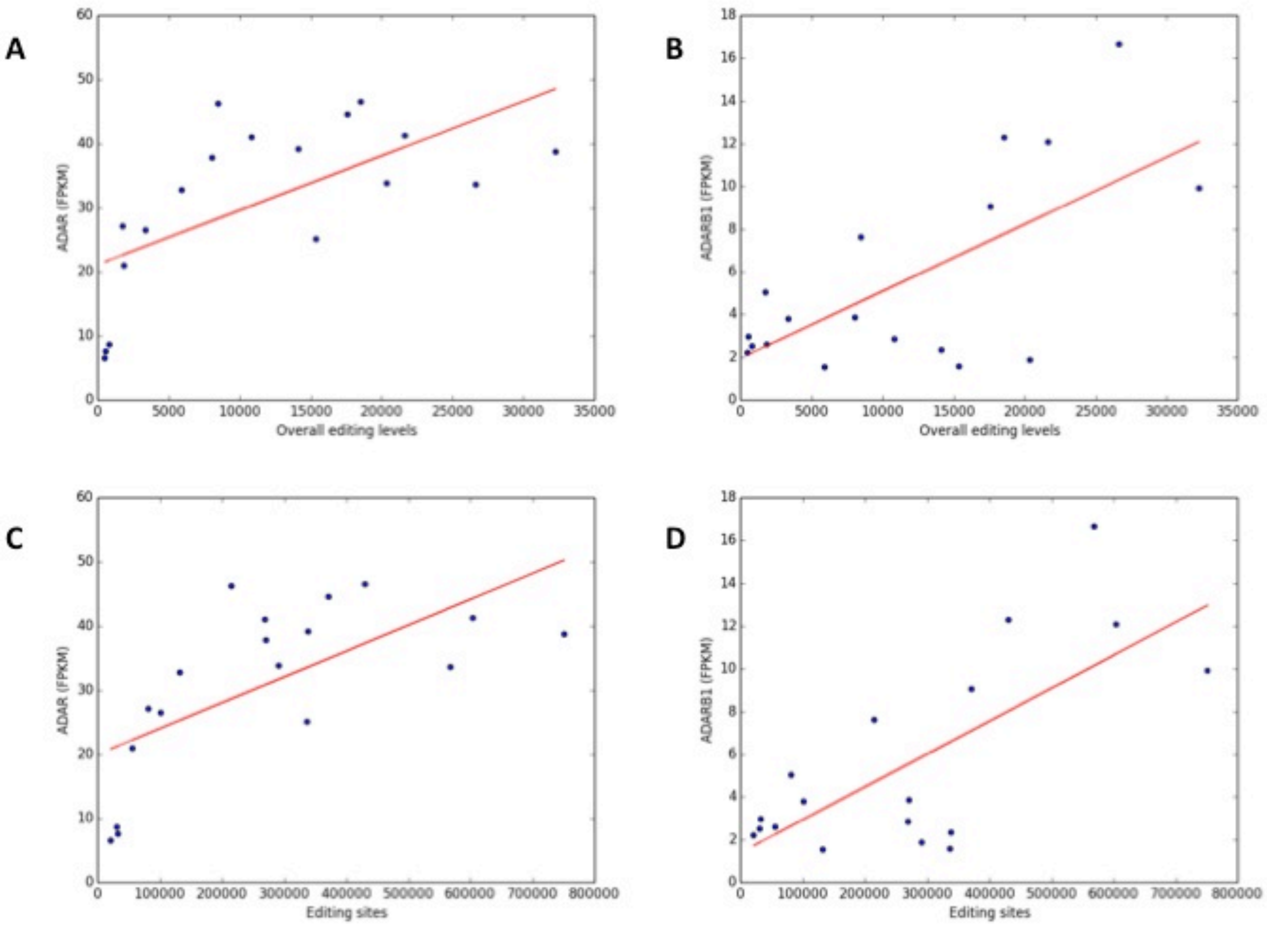

Supplementary Figure 15. RNA editing Vs expression of ADARs in all samples. In A) we correlate overall RNA editing levels and ADAR expression values in all tissues and individuals (n=18). In B) we correlate overall RNA editing levels and ADARB1 expression values in all tissues and individuals (n=18). In C) and D) we repeat calculations to correlate the number of detected RNA editing sites and expression values of ADAR and ADARB1, respectively (n=18). Spearman rank correlation coefficients (rho) and P_values are shown in each graph.

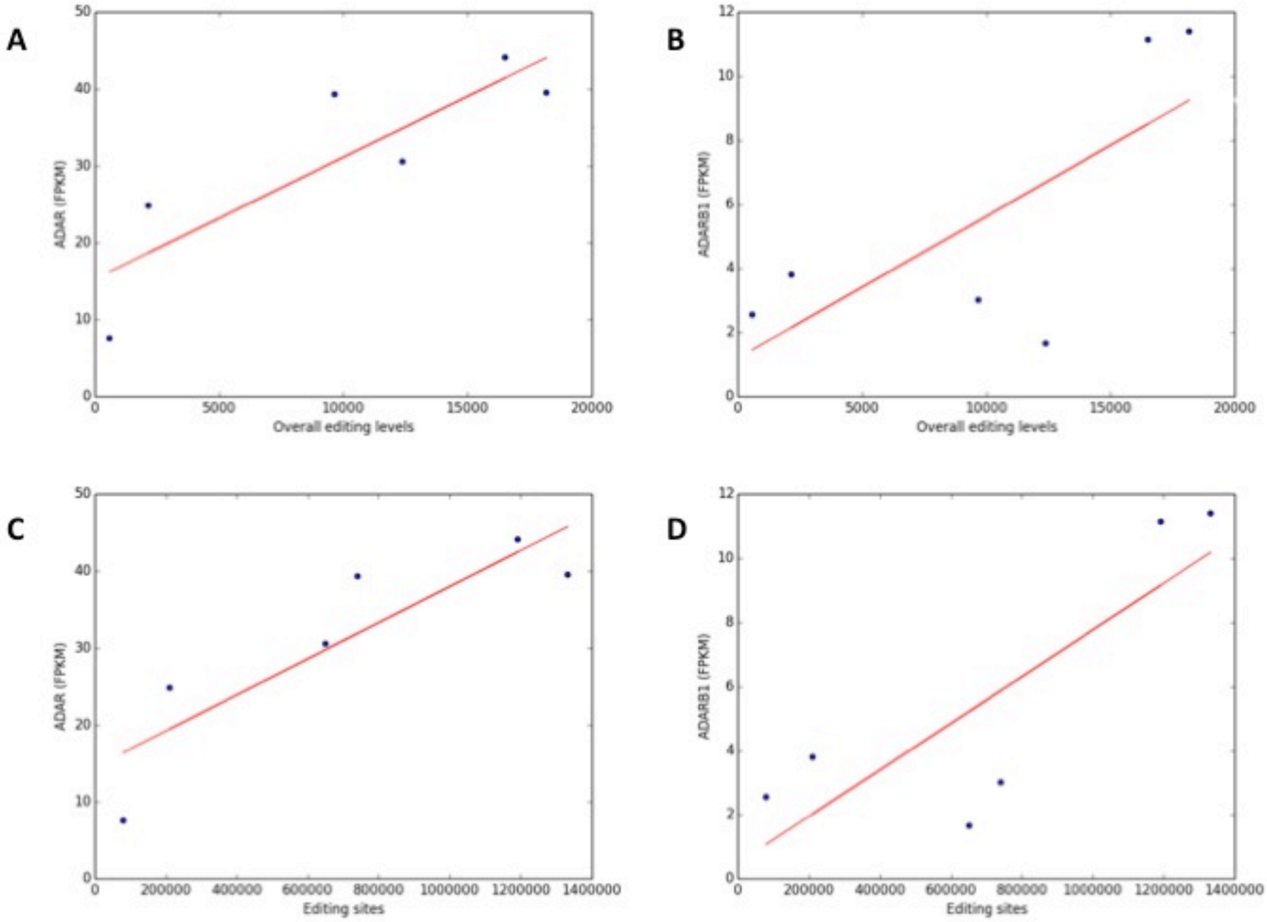

Supplementary Figure 16. RNA editing Vs expression of ADARs in tissue groups. In A) we correlate overall RNA editing levels and ADAR expression values in all tissue groups (n=6). In B) we correlate overall RNA editing levels and ADARB1 expression values in all tissue groups (n=6). In C) and D) we repeat calculations to correlate the number of detected RNA editing sites and expression values of ADAR and ADARB1, respectively (n=6). Spearman rank correlation coefficients (rho) and P_values are shown in each graph.

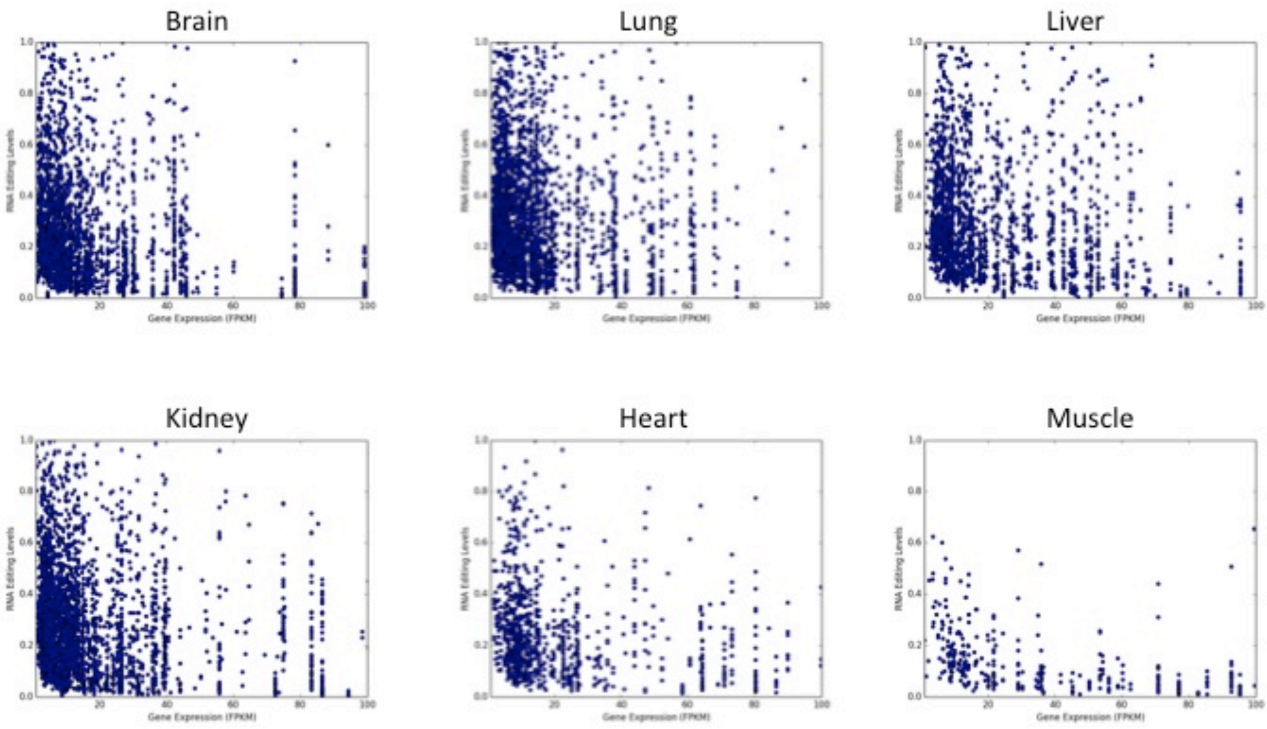

Supplementary Figure 17. Scatter Plot of RNA editing levels and gene expression values in all tissue groups.

Supplementary Tables

All Supplementary Tables can be downloaded from

https://www.dropbox.com/s/5shbtvlzpnqtyx2/Supplementary-Tables.zip?dl=0 or required to Ernesto

Picardi ernesto.picardi@uniba.it.

Supplementary Table 1. Main statistics about samples and high throughput sequencing data used in this study. The legend is included in the spreadsheet.

Supplementary Table 2. Pairwise comparison of inosinomes in tissues and individuals using as discriminative variable the number of edited genes.

Supplementary Table 3. Average numbers of RNA editing events detected in this study and in a recent work based on GTEx experiments (GIREMI). For each tissue we report also the number of sequenced RNA fragments used to call A-to-I events (NA: data not available).

Supplementary Table 4. Table of editing positions residing in precursors or mature miRNAs detected in RNA-Seq data.

Supplementary Table 5. Number of tissue specific editing events and genes.

Supplementary Table 6. Gene enrichment in edited tissue specific genes.

Supplementary Table 7. List of positions with differential RNA editing. Column labels are indicated in the first row.

Supplementary Table 8. Gene enrichment in DisGenet database.
